# Restoring microenvironmental redox and pH homeostasis inhibits neoplastic cell growth and migration: therapeutic efficacy of esomeprazole plus sulfasalazine on 3-MCA-induced sarcoma

**DOI:** 10.18632/oncotarget.18713

**Published:** 2017-06-27

**Authors:** Enrica Balza, Patrizia Castellani, Paola Sanchez Moreno, Patrizia Piccioli, Iria Medraño-Fernandez, Claudia Semino, Anna Rubartelli

**Affiliations:** ^1^ Cell Biology Unit, IRCCS AOU San Martino – IST, 16132 Genoa, Italy; ^2^ Protein Transport Unit, Division of Cell and Molecular Biology, San Raffaele Institute, 20132 Milan, Italy; ^3^ Present address: Nanobiointeractions and Nanodiagnostics, Istituto Italiano di Tecnologia, 16163 Genoa, Italy

**Keywords:** anti-oxidants, 3-methylcholanthrene-induced tumorigenesis, proton pump inhibitors, tumor-associated macrophages, xCT

## Abstract

Neoplastic cells live in a stressful context and survive thanks to their ability to overcome stress. Thus, tumor cell responses to stress are potential therapeutic targets. We selected two such responses in melanoma and sarcoma cells: the xc- antioxidant system, that opposes oxidative stress, and surface v-ATPases that counteract the low pHi by extruding protons, and targeted them with the xc- blocker sulfasalazine and the proton pump inhibitor esomeprazole. Sulfasalazine inhibited the cystine/cysteine redox cycle and esomeprazole decreased pHi while increasing pHe in tumor cell lines. Although the single treatment with either drug slightly inhibited cell proliferation and motility, the association of sulfasalazine and esomeprazole powerfully decreased sarcoma and melanoma growth and migration. In the 3-methylcholanthrene (3-MCA)-induced sarcoma model, the combined therapy strongly reduced the tumor burden and increased the survival time: notably, 22 % of double-treated mice recovered and survived off therapy. Tumor-associated macrophages (TAM) displaying M2 markers, that abundantly infiltrate sarcoma and melanoma, overexpress xc- and membrane v-ATPases and were drastically decreased in tumors from mice undergone the combined therapy. Thus, the double targeting of tumor cells and macrophages by sulfasalazine and esomeprazole has a double therapeutic effect, as decreasing TAM infiltration deprives tumor cells of a crucial allied. Sulfasalazine and esomeprazole may therefore display unexpected therapeutic values, especially in case of hard-to-treat cancers.

## INTRODUCTION

The excess of Reactive Oxygen Species (ROS) and the consequent oxidative stress are traditionally considered to cause cancer [1]. However, it has recently been proposed that tumor development and progression are rather promoted by antioxidants. Although upregulated to detoxify ROS, antioxidant levels exceed those required to re-establish redox equilibrium and exert pro-tumor functions, including inhibition of apoptosis, increased cell proliferation, resistance to therapy [2]. This view was built on earlier observations that human primary cancers display a highly reduced redox state both in neoplastic cells and infiltrating inflammatory cells, and that the levels of antioxidants correlate with tumor aggressiveness both *in vitro* and *in vivo* [3]. Later studies confirmed and extended these findings [4, 5]. An antioxidant system particularly important in tumors is the cystine-cysteine redox cycle xc-. This system is composed by a membrane bound heterodimer where the specific light chain, xCT, mediates the uptake of cystine, the oxidized form of cysteine that prevails extracellularly, in exchange with glutamate [6]. After intracellular reduction by members of the thioredoxin family [7], cysteine is employed in protein and glutathione biosynthesis, and in part released outside, thus causing a reduction of intra and extracellular redox state [2, 3]. Not only cysteine but also oxidoreductases such as thioredoxin, overexpressed in tumors, may be externalized [8] and contribute to the functional switch of extracellular protein activity by remodeling redox-sensitive disulfides [9–11]. A reducing microenvironmental redox state also increases cancer cell invasive ability [12].

xc- is upregulated in many tumor types [3, 13, 14] and induced by treatment with pro-oxidant drugs, contributing to drug resistance[13, 15]. Notably, it is highly expressed in cancer stem cells [15, 16], and is repressed by p53 [17]. Sulfasalazine, a non-toxic drug largely used in clinics, is a strong inhibitor of xc- [18] and has provided promising results in preclinical studies especially in association with classic anti-tumor drugs [14, 19].

Tumor cells must also face the stress derived from the metabolic shift to glycolysis [20] with the consequent production of acidic metabolites that, if not extruded, would kill cells. Upregulation of enzymes such as carbonic anhydrase IX [21] and of transporters such as v-ATPases, NHE, MCTs, allows cancer cells to extrude protons and eliminate lactic or carbonic acid [22], with a double advantage: on the one hand, cells maintain a pHi compatible with life; on the other hand, a concurrently extracellular acidification occurs that facilitates tumor progression through various mechanisms [22]. Thus, interfering with pH regulation in tumors has been proposed as a novel anti cancer strategy [23]. v-ATPases are normally restricted to intracellular acidic organelles, but translocate to the plasma membrane in tumor cells representing a potential therapeutic target [22, 24]. Proton pump inhibitors (PPI), that block the gastric H^+^/K^+^ ATPase pump, also inhibit v-ATPases [25, 26] exerting anti-tumor effects [22, 24]. Remarkably, PPI and carbonic anhydrase IX inhibitors have been shown to sinergize in inhibiting proliferation and inducing cell death in melanoma cells [27].

A hallmark of most tumors is the presence of abundant TAM. The majority of TAM display M2 phenotype and exert pro-tumor activities [28]. Interestingly, activated monocytes/macrophages share with tumor cells both the upregulation of xCT [14, 29] that occurs in response to ROS induced in inflammatory cells by PRR triggering [29] and the membrane expression of v-ATPases [30, 31], likely due to the need to extrude protons, as also activated macrophages undergo metabolic shift to aerobic glycolysis [32].

In preclinical studies, treatment with sulfasalazine or esomeprazole sensitizes cells to chemotherapeutic drugs increasing their effectiveness [23, 33–39]. We then investigated whether the combination of sulfasalazine and esomeprazole, both drugs devoid of toxic effects, is advantageous over the use of each of them with chemotherapeutics. Our results indicate that sulfasalazine and esomeprazole synergically inhibit cell growth and migration of melanoma and sarcoma cells. In particular, in the experimental model of 3-MCA -induced mouse sarcoma, the combined treatment strongly delays the sarcoma growth, decreases the tumor size and increases survival. These effects specifically correlate with a dramatic reduction of TAM.

## RESULTS

### Primary human tumors are more acidic and express more antioxidants than their normal counterparts

**Figure 1:**
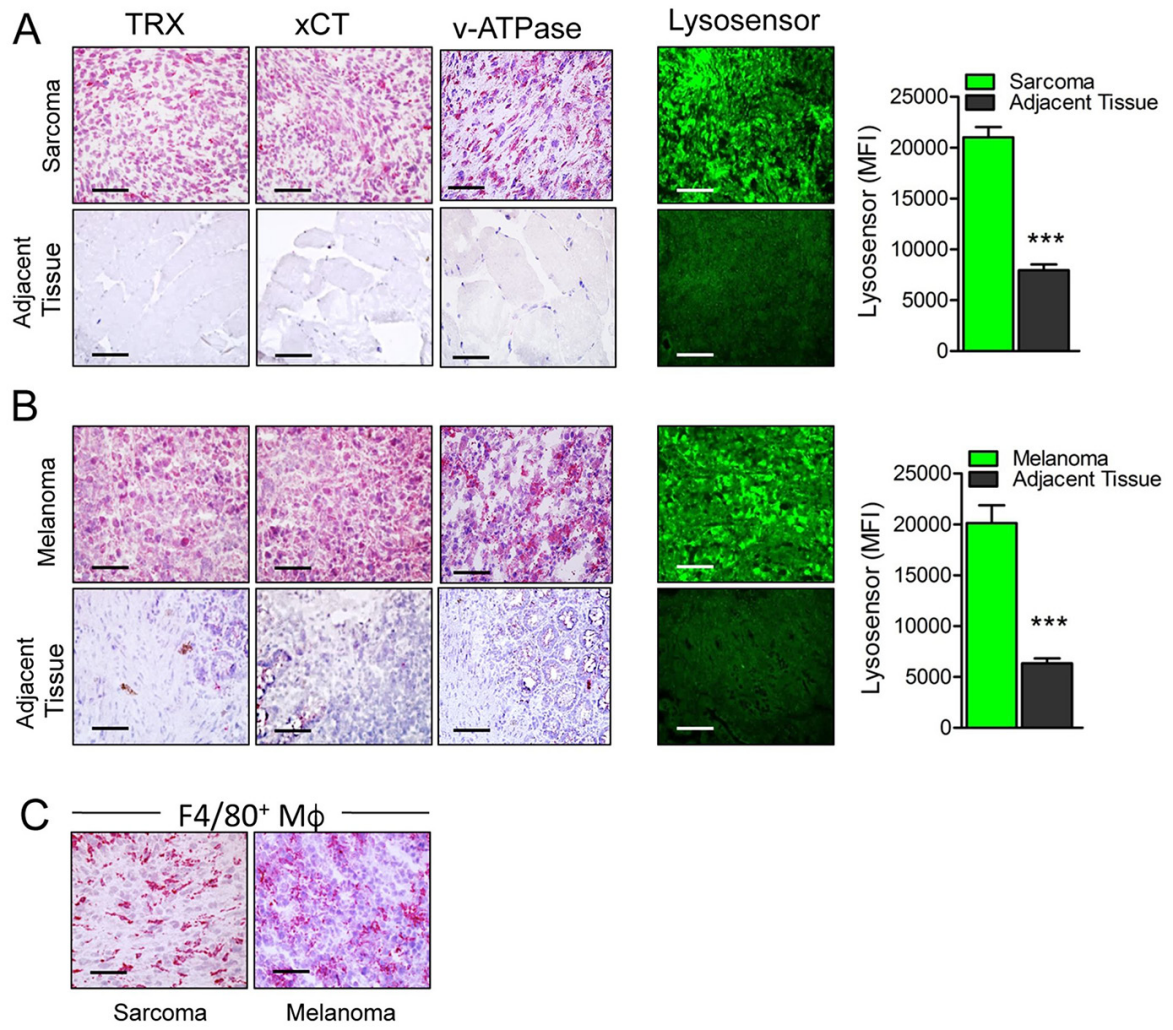
Primary human tumors are more acidic and display higher levels of antioxidants than their normal counterparts **(A, B)** Serial cryostat sections of frozen samples from human sarcoma (A) and melanoma (B) and normal adjacent tissues stained with anti Thioredoxin (TRX), anti xCT, anti v-ATPase or LysoSensor Green DND-189 as indicated. Quantification of the LysoSensor fluorescence levels in sarcoma (A) and melanoma (B) and normal adjacent tissues is indicated. Results are expressed as mean fluorescence intensity (MFI) obtained in 20 chosen fields ± SEM. ***P ≤ 0.001. **(C)**, Serial cryostat sections of frozen sample from human sarcoma and melanoma stained with anti-F4/80. Scale bar, 30μm. One representative case of sarcoma and melanoma out of 5 performed is shown.

To verify overexpression of antioxidants and low pH in sarcoma and melanoma, surgical samples of human primary or metastatic sarcoma (n=10) and melanoma (n=10) were cut and immediately analyzed for the expression of xCT, thioredoxin and v-ATPase by immunohistochemistry, and for the presence of acidic pH using the pH dependent LysoSensor dye. Both sarcoma (Figure 1A) and melanoma (Figure 1B) samples were highly positive for xCT, thioredoxin and v-ATPase in strong contrast with the adjacent normal tissues that were almost negative, confirming previous results [3, 14, 15]. Tumor samples were also significantly more acidic than the adjacent tissues (Figure 1A and 1B, right panel and graphs). The specificity of LysoSensor fluorescence was tested by pre-incubating tumor sections with buffer at pH 8.8 before staining. This treatment strongly inhibited LysoSensor fluorescence intensity (Supplementary Figure 1). Moreover, in agreement with previous studies [28], all samples displayed a strong infiltrate of TAM (Figure 1C).

These data represented a proof of principle for testing anti-acid and anti-antioxidant compounds as anti-tumor drugs.

### Treatment with (S)-4carboxyphenylglycine (sCPG) and esomeprazole strongly decreases cysteine release and partially restore physiologic pHi and pHe

The activities of sCPG as an xCT inhibitor and of esomeprazole as a blocker of proton extrusion were tested on the following tumor cell lines: a murine sarcoma cell line, MCA obtained from a 3-MCA tumor [14], the commercial murine melanoma cell line B16-F10 (from here: B16), and the human melanoma MePa cells, obtained from a human melanoma metastasis [40]. sCPG was used instead of sulfasalazine as sulfasalazine interferes with the assay for cysteine release [13].

**Figure 2:**
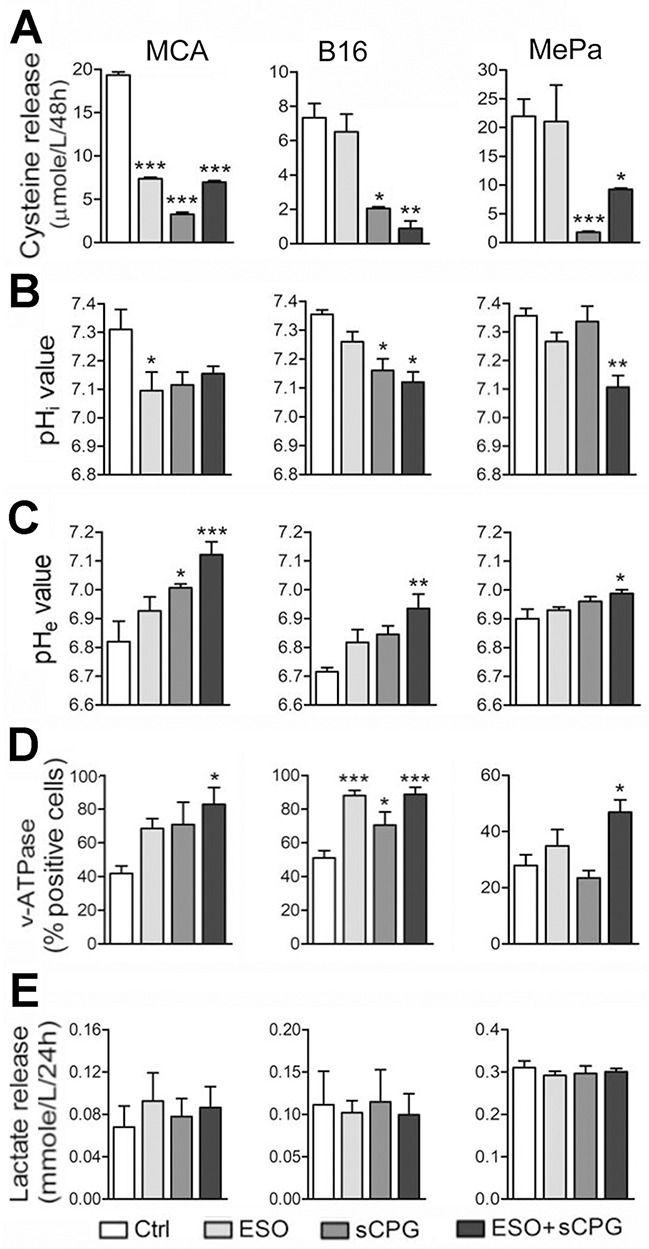
sCPG inhibits cysteine release by tumor cells and esomeprazole modulates pHi and pHe **(A-E)** Data shown in left panels refer to MCA cells; in middle panels to B16 cells; in right panels to MePa cells. (A), Cysteine release by MCA, B16 and MePa cell lines, quantified after 48 h of treatment with esomeprazole (ESO), sCPG and ESO+sCPG. (B), Value of cytosolic pH (pHi) evaluated by cytofluorimetry in the three cell lines treated for 48h with the different drugs and loaded with the pH-sensitive fluorescent probe BCECF-AM. (C), Value of pHe in the three cell lines treated as in (A). (D), FACS analysis of surface expression of v-ATPases in MCA, B16 and MePa cell lines treated as in (A). (E), Lactate release detected in cell supernatants 24 h after treatment with ESO, sCPG and ESO+sCPG. Data are expressed as indicated in each panel (mean ± SEM of at least 3 experiments). Statistical significance was estimated between drug-treated and untreated cells. *P < 0.05, **P < 0.01, ***P < 0.001.

As shown in Figure 2A all the three cell lines release different but consistently high levels of free cysteine, in agreement with the expression of relevant levels of xCT and thioredoxin (Supplementary Figure 2), two key components of the xc- system [2]. In all cases, cysteine release was blocked by sCPG (Figure 2A) whereas esomeprazole alone partially inhibited cysteine release by MCA cells but not by B16 and MePa cells (Figure 2A). Staining with the BCECF-AM probe that measures changes in cytosolic pH showed that esomeprazole treatment decreased the pHi with different efficacy in the three cell lines (Figure 2B). A pHi decrease was also observed with sCPG. Notably, the co-treatment with esomeprazole and sCPG caused a further drop of pHi in B16 and, at a greater extent, in MePa cells (Figure 2B). The decrease of pHi following treatment with esomeprazole alone or with sCPG was paralleled by a raise of pHe (Figure 2C).

The three cell lines did not express the gastric H^+^/K^+^ proton pump that is the primary molecular target of proton pump inhibitors (PPIs; not shown), but displayed significant amounts of surface v-ATPases (Figure 2D) that are targeted by PPIs on tumor cells [25–26]. After 24 hours of culture with the two drugs, v-ATPase staining increased in cells exposed to esomeprazole and, at a greater extent, to esomeprazole plus sCPG (Figure 2D).

Although esomeprazole or esomeprazole plus sCPG actually increased pHe, they were unable to restore the physiologic extracellular pH. A possible explanation is that neoplastic cells are endowed with other transporters and enzymes that contribute to modulate the pH [21–23] and that different acidic compounds including lactic acid are released by neoplastic cells. In fact, the three cell lines, like most neoplastic cells [22, 23], released elevated levels of lactate that were unaffected by the drugs, alone or in combination (Figure 2E). In support to the involvement of different acidic catabolites in the generation of low pHe, the highest levels of lactic acid are released by MePa cells whose pHe is only slightly affected by esomeprazole and esomeprazole plus sCPG treatment.

### The combined exposure to sCPG and esomeprazole significantly inhibits *in vitro* tumor cell proliferation

**Figure 3:**
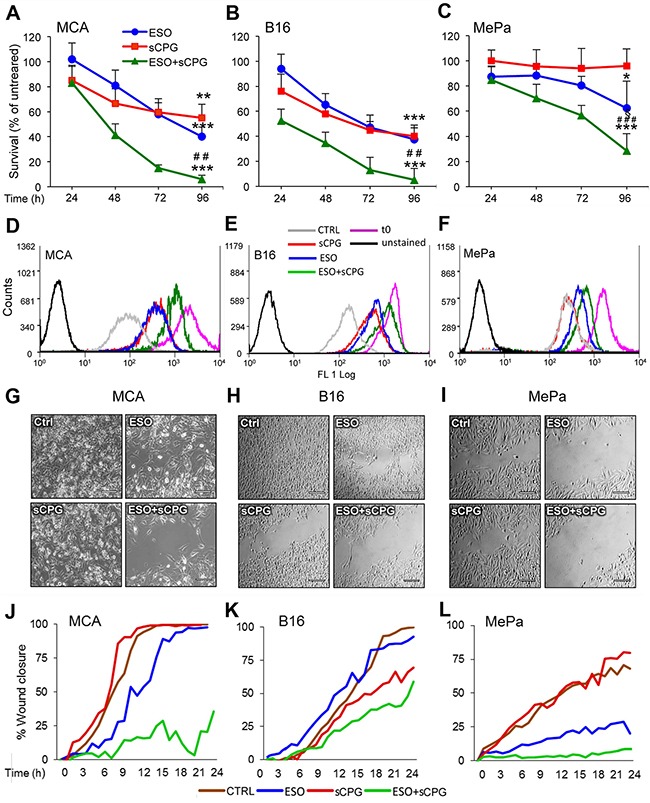
Esomeprazole plus sCPG impair tumor cell growth, proliferation and migration **(A-C)**, Survival rate of MCA (A), B16 (B) and MePa (C) cells treated with ESO (blue circles) and sCPG (red squares), alone or in combination (green triangles) for 24 h, 48 h, 72 h and 96 h was determined by Cristal Violet assay. Data are expressed as percent of untreated cells (mean of 3 experiments ± SEM). Significance of the differences in survival of drug-treated vs untreated cells is shown by asterisk (*P < 0.05, **P < 0.01, ***P < 0.001); significance of the differences in survival of double treated cells vs single ESO or sCPG-treated cells is shown by number sign (^##^P < 0.01; ^###^P < 0.001). **(D-F)**, Proliferation rate of cells cultured for 72 h without (gray) or with ESO (blue), sCPG (red) or ESO+sCPG (green) was determined by CFSE assay. The profile of CSFE-loaded cells at t0 is shown in purple; the profile of unstained cells is shown in black. One representative experiment out of 3 is shown. **(G-I)**, Migration of cells untreated (Ctrl) or pretreated 48 h with ESO and sCPG alone or in combination were analyzed in a gap filling assay (see Materials and Methods). Images show frames of a representative video, taken after 16 h 40min (MCA) or 24 h (B16 and MePa) of culture at 37°C. Scale bar corresponds to 200μm. **(J-L)**, Results are represented as percentage of gap closure against time. One representative experiment out of 2 performed is shown.

Having established the molecular effects of sCPG and esomeprazole on neoplastic cells, we investigated their anti-tumor efficacy. *In vitro*, the growth/survival of the 3 cell lines was slightly affected by esomeprazole and sCPG alone, with different sensibility (Figure 3A–3C). In particular, whilst MCA and B16 cells were sensitive to both drugs even when provided individually, with a decrease in cell number of about 40% at day 3, esomeprazole and sCPG only marginally affected the survival of MePa cells. (Figure 3A–3C). However, the association of the two drugs had a dramatic effect in all the 3 cell lines, with a decreased in cell survival of more than 85% in MCA and B16, and more than 45% in Me-Pa cells. After 4 days of treatment, very few viable cells were found in the wells treated with esomeprazole plus sCPG (Supplementary Figure 3). The same experiments performed with sulfasalazine induced identical effects as sCPG on cell survival (not shown), confirming that the two xCT inhibitors have the same anti-tumor efficaciousness [41]. Monitoring cell proliferation by the CFSE assay [42] verified that the combination of esomeprazole and sCPG was highly effective in slowing down the proliferation of the three tumor cell lines (Figure 3D–3F). Within 96 hours, MCA and B16 cells treated with esomeprazole plus sCPG replicated about 5 times less than untreated cells, whereas the inhibitory effect on proliferation was less evident in MePa cells (50% less proliferation in esomeprazole plus sCPG treated cells, with respect to untreated cells) (Supplementary Figure 4).

Cisplatin is used in many tumors, including melanoma [43], and is one of the drug of choice for sarcoma [44]. However, even in combination with other chemotherapeutics, cisplatin is often unable to eliminate the tumor; moreover, it causes severe side effects [45, 46]. In agreement with previous studies on different models [33–35, 38], sulfasalazine and esomeprazole sensitized the three tumor cell lines to cisplatin (Supplementary Figure 5). However, in all cases the combination of esomeprazole plus sCPG was equally or more efficacious than the association of either drug with cisplatin: we then focused on the association of sulfasalazine (or sCPG) and esomeprazole as an alternative anticancer combined therapy.

### Esomeprazole plus sCPG strongly slow down cell migration

Tumor cell migration is essential for metastasis formation. To study the effects of esomeprazole and sCPG on the migration potential, we used gap-filling assays that measure the combined rates of cell division and motility [47] of MCA, B16 and MePa cells. After 48 hours of pretreatment with the esomeprazole and sCPG, alone or in combination, an equal number of untreated or treated cells were seeded in the proper wound healing plates and migration was monitored in real time by microscopy for 24 hours (Figure 3G–3L and Supplementary Videos 1-8). The cell lines display different speed in closing the gap: the sarcoma cells (Figure 3G and 3J, Supplementary Videos 1-4) are the most rapid and the human melanoma (Figure 3I and 3L, Supplementary Videos 5-8) the slowest. sCPG alone inhibited migration in B16 cells, at to a lesser extent in MePa cells but did not impair MCA cell migration that was instead delayed by esomeprazole. In all cases the association of esomeprazole and sCPG was significantly more powerful: by the time untreated cells closed the gap, >50% of the gap was still open in B16 and >80% in MCA and MePa double-treated cells. Remarkably, double-treated cells exhibited not only a reduced speed of migration but also a slower rate of cell division, without relevant occurrence of cell death as observed in Supplementary Videos. Identical results were obtained using sulfasalazine in place of sCPG (Supplementary Figure 6)

### Esomeprazole plus sulfasalazine significantly inhibit tumor cell growth *in vivo*

**Figure 4:**
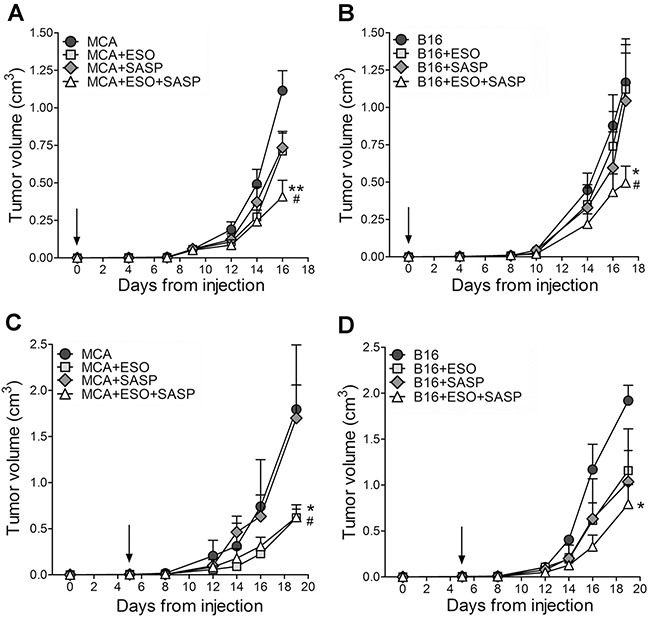
Esomeprazole plus sulfasalazine treatment reduces tumor growth *in vivo* Mice, s.c. injected with MCA **(A, C)** or B16 cells **(B, D)**, were left untreated or were treated with ESO, sulfasalazine (SASP) or ESO plus SASP, 5 h after tumor cell injection (A, B, n=12 mice per each treatment group) or when the tumor became palpable (C, D n=4 mice per each treatment group). Arrows indicate the day in which treatments started. Tumor volumes of MCA (A, C) and B16 (B, D) untreated (circles) or treated with ESO (squares), SASP (diamonds) or ESO+SASP (triangles) were measured over time and results are expressed as cm^3^ (mean ± SEM). Statistical significance of tumor volume differences in double treated vs untreated mice is shown by asterisk (*P < 0.05, **P < 0.01). Statistical significance of tumor volume differences in double treated mice vs single treated mice (with SASP in the case of MCA tumors; with ESO or SASP in the case of B16 tumors) is shown by number sign (^#^P < 0.05).

The efficacy of esomeprazole and sulfasalazine was then studied *in vivo*. First, MCA and B16 cells were inoculated into the flank of syngeneic mice that were left untreated or treated with the two drugs alone or in combination (Figure 4). Both MCA and B16 tumors grew fast in untreated mice that were sacrificed when the volume reached 1,2-1,5 cm^3^.

In a first series of experiments, the administration of esomeprazole and/or sulfasalazine was started 5 hours after tumor cell inoculation. The single drugs inhibited tumor cell growth to some extent in MCA- (Figure 4A) but not in B16-injected mice (Figure 4B). In contrast, the association of esomeprazole and sulfasalazine strongly inhibited tumor growth, resulting in a mean tumor volume about 60% smaller in double treated mice compared to untreated mice, in both MCA and B16 injected mice (Figure 4A and 4B).

We then evaluated the therapeutic efficacy of esomeprazole and sulfasalazine starting administration when the tumor became palpable, which in most experiments occurred at day 5 from tumor cell injection, both in MCA and B16 tumors (Figure 4C and 4D). Remarkably, even if provided later, the combined therapy resulted in an inhibition of tumor growth by more than 60% compared to untreated mice. While esomeprazole alone provided similar results as the double treatment in MCA injected mice (Figure 4C), the association of esomeprazole and sulfasalazine strongly increased the therapeutic efficacy of the single drugs in B16-injected mice (Figure 4D).

### Treatment with esomeprazole plus sulfasalazine impairs tumor growth and increases survival in the 3-MCA-induced tumorigenesis

**Figure 5:**
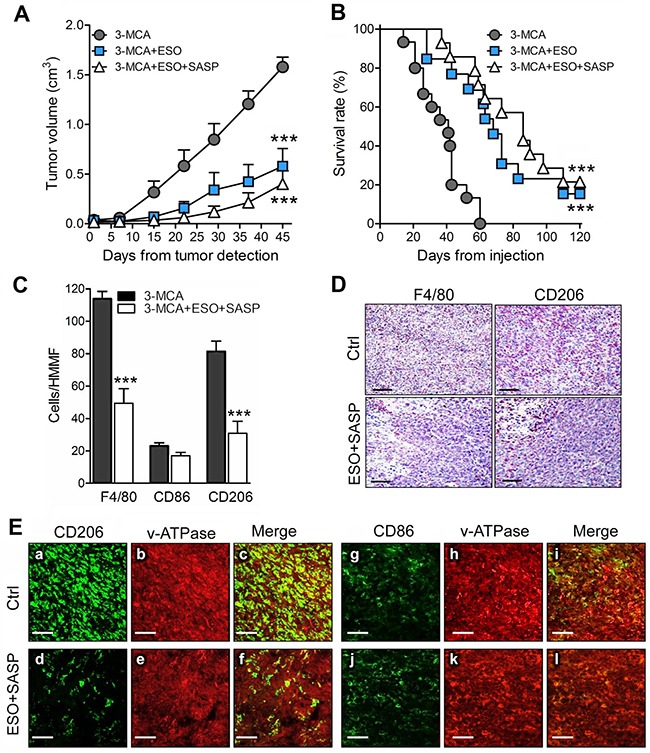
The association of esomeprazole plus sulfasalazine is highly therapeutic in the 3-MCA-induced sarcoma **(A, B)**, Mice were injected s.c with 3-MCA and untreated or treated with ESO or ESO+SASP. For each mouse, the day on which the tumor was first detected is defined as day 0. Fifteen mice per group were treated. (A) Results of tumor volume measurement are expressed as cm^3^ (mean ± SEM). (B), Survival was monitored up to 120 days. (A and B): ***P ≤ 0.001 (ESO and ESO+SASP treated vs untreated mice). **(C)**, Quantificationof F4/80^+^ (total TAM) CD86^+^ (M1) and CD206^+^ (M2) macrophages on serial cryostat sections of 3-MCA tumors from untreated (Ctrl) or ESO+SASP-treated mice. The results are expressed as cell number per HMMF (mean ± SEM). Twenty HMMF from 3 tumors for each group of treatment were counted. ***P ≤ 0.001. **(D)**, One representative immunostaining (out of 5) of F4/80^+^ and CD206^+^ macrophages on serial cryostat section of 3-MCA tumors from untreated (Ctrl) or ESO+SASP-treated mice. **(E)**, Double immunofluorescence analysis with anti-CD206 or anti-CD86 (green) and anti-v-ATPase (red), performed on cryostat sections from 3-MCA tumors obtained from untreated (Ctrl; a-c, g-i) and ESO+SASP (d-f, j-l) treated mice. Scale bar, 30μm. One representative experiments out of 3 performed is shown.

The positive effects of the combined therapy on the tumor cell lines prompted us to investigate the therapeutic efficacy of esomeprazole plus sulfasalazine in a mouse model of multi-step tumorigenesis, namely, the 3-MCA-induced sarcoma. In the same experimental model we previously showed that mice treated with sulfasalazine alone developed tumors than were only slightly smaller than in untreated mice, and did not display relevant changes in the number of infiltrating macrophages [14]. In the present trial, 60 mice were injected with 3-MCA. Of these, 75% developed a tumor after 45 to 60 days. When the tumor became palpable, 15 mice per group were untreated or treated with esomeprazole or esomeprazole plus sulfasalazine for 60 days as detailed in Materials and Methods. At day 45 from the beginning of the treatment, the mean tumor volume was 60% smaller in esomeprazole -treated mice and 75% smaller in esomeprazole plus sulfasalazine treated-mice compared to untreated mice (Figure 5A). Similarly, the survival curves show that while untreated mice died within day 60, mice treated with esomeprazole and esomeprazole plus sulfasalazine significantly increased the survival rate (Figure 5B). Notably, 15% of esomeprazole and 22% of esomeprazole plus sulfasalazine -treated mice were still alive at day 120, two months after the end of the treatment.

An abundant infiltrate of TAM (F4/80^+^) was detected in tumors from untreated mice with a strong predominance of CD206^+^ M2 over CD86^+^ M1 macrophages (Figure 5C). The combined treatment with esomeprazole and sulfasalazine resulted in a dramatic depletion of M2 macrophages, while the M1 TAM were only slightly decreased (Figure 5C). The few TAM found in treated tumors were restricted to the necrotic areas (Figure 5D).

Macrophages are known to express membrane v-ATPases [30, 31], the putative target for esomeprazole [24, 26]. Confocal analysis of tumor sections, double stained with anti-CD206 or anti-CD86 and anti-v-ATPases, revealed that both tumor cells and TAM expressed v-ATPases, the fluorescence intensity being much higher in macrophages (Figure 5E, panels a-c). The co-distribution of v-ATPases and CD206 or CD86 was very high, both in tumors from untreated and esomeprazole plus sulfasalazine-treated mice, even if in treated tumors the amount of CD206+ TAM was highly decreased (compare panels a-c with d-f).

**Figure 6:**
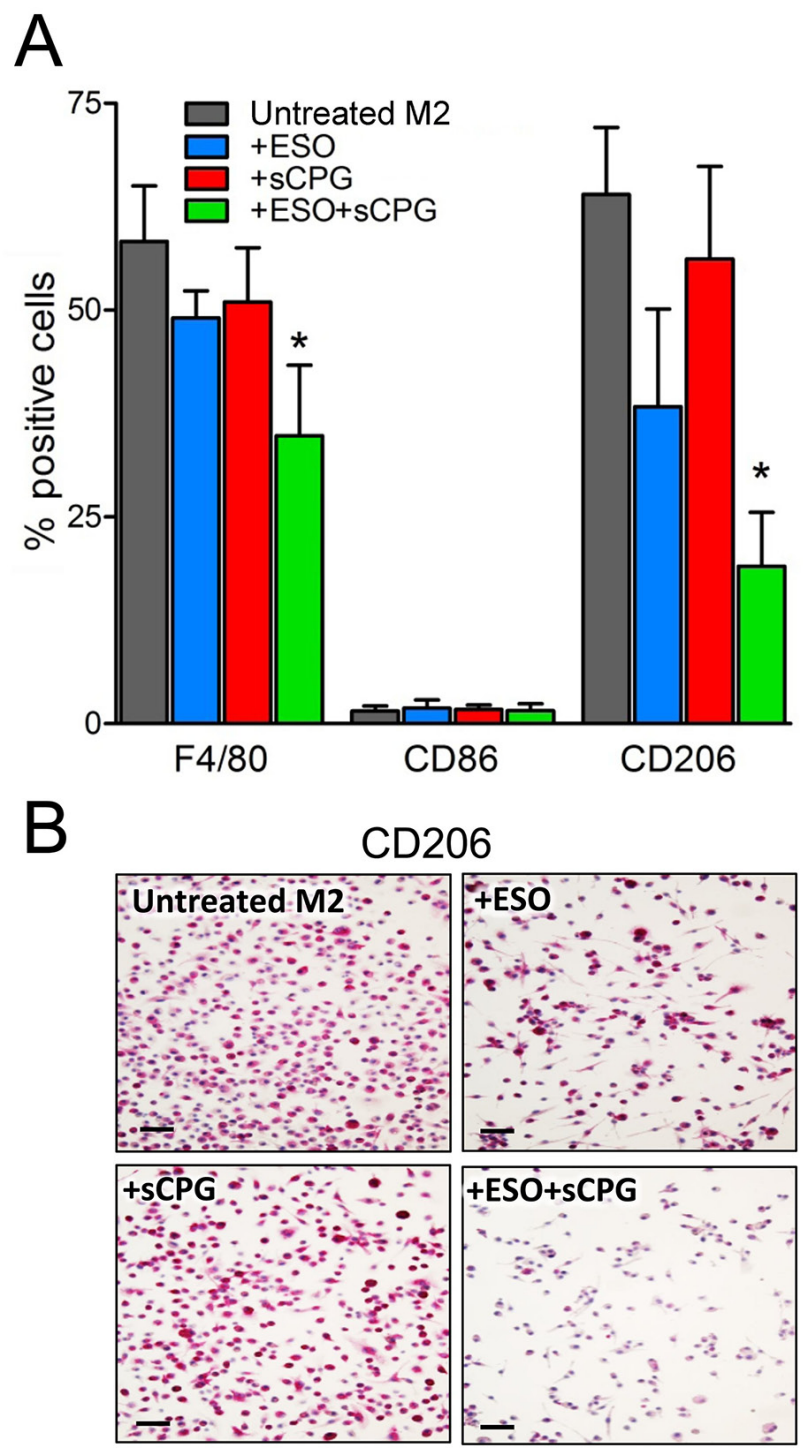
The association of esomeprazole plus sCPG reduces M2 polarized BMDM *in vitro* **(A)**, BMDM were polarized to the M2 phenotype by exposure to IL-4 for 48 h, in the absence or presence ESO, sCPG and ESO+sCPG, and then analyzed by flow cytometry for the expression of surface markers of total (F4/80^+^), M1 (CD86^+^) and M2 (CD206^+^) macrophages. Results are expressed as percentage of positive cells (mean ± SEM, n=4). *P < 0.05 vs untreated M2. **(B)**, Immunohistochemical assessment of CD206 expression by M2 polarized BMDM untreated or treated with ESO, sCPG and ESO+sCPG. One representative experiments out of 3 is shown. Scale bar, 30μm.

To investigate whether the two drugs target M2 TAM directly, or rather by affecting tumor cells or tumor microenvironment, we tested their effects on *in vitro* derived macrophages (Figure 6). Murine bone marrow derived macrophages (BMDM), grown in the presence of granulocyte macrophage colony stimulating factor (GM-CSF), were polarized to the M2 phenotype by 48 hours of exposure to IL-4 with or without esomeprazole and/or sCPG. In the absence of drugs, 60-70% of the cells were F4/80^+^, and displayed the M2 marker CD206. As expected, under these conditions the percent of cells expressing the M1 marker CD86 was irrelevant. Single treatment with esomeprazole or sCPG did not significantly change the percent of F4/80^+^ and CD206^+^ cells. In contrast, the association of esomeprazole and sCPG resulted in a decrease of F4/80^+^ and, at a greater extent, of CD206^+^ macrophages with no concomitant increase of cells displaying the CD86 marker. Taken together these data indicate that the combined treatment directly targets M2 macrophages.

## DISCUSSION

In this study we demonstrate that the concomitant targeting of two stress responses, evolved by tumor cells to survive oxidative stress and intracellular acidosis, has strong anti-tumor effects and may represent a novel efficacious therapeutic approach.

The observation that primary human sarcoma and melanoma overexpress xCT, the functional component of the xc- antioxidant system, and membrane v-ATPases that mediate proton extrusion, represented a proof of principle for testing anti-antioxidant and anti-acid compounds as anti-tumor drugs. We used sulfasalazine (or sCPG) to block xCT and esomeprazole to inhibit membrane v-ATPases.

*In vitro* studies demonstrated that the two drugs supplied alone efficiently inhibited their molecular targets, but exhibited a modest effect on tumor cell behavior. In contrast, the combined administration of both drugs displayed dramatic anti-tumor effects. In particular, esomeprazole plus sCPG treatment profoundly decrease the rate of tumor cell proliferation *in vitro* and slow down cell migration, with drug-treated cells appearing sluggish and slow-moving in gap filling experiments (Supplementary Videos 1-12). The occurrence of dying cells among double treated tumor cells is infrequent, representing an additional advantage of these combined therapy, as massive tumor necrosis is often detrimental and can be fatal [48].

Thus, while blocking a single defense mechanism only partially affect the neoplastic cell behavior, the simultaneous inhibition of two different stress responses strongly weakens tumor cells.

Both esomeprazole and sulfasalazine have been reported to sensitize cells to cisplatin [13, 18, 35–39, 50], that is a first line drug for many human tumors, including sarcoma [44]. Our data show that the combination of esomeprazole and sCPG is equally or more efficient in inhibiting tumor cell growth than the association of esomeprazole or sCPG with cisplatin. Thus, the combined therapy we propose in this study is advantageous over the use of each inhibitor with chemotherapeutics, since, unlike classical anti-tumor drugs, esomeprazole and sulfasalazine do not display toxic effects that may be highly invalidating for the patient [44, 45].

*In vivo*, the combined therapy is dramatically more efficacious than the single drugs in the model of syngeneic transplantation of MCA and B16 tumor cells in mice, indicating that also *in vivo* the simultaneous inhibition of xCT and proton extrusion is intolerable for tumor cells. In *in vivo* experiments, sulfasalazine was preferred to sCPG because it displays reduced nociceptive behaviors and an extended time until the onset of evidence of pain, compared to sCPG [41].

We have previously reported that, in the 3-MCA induced sarcoma model, sulfasalazine, and at a greater extent sulfasalazine in combination with ibuprofen, inhibit tumor growth and improved survival [14]. In that study however no mice were alive at 120 days from the beginning of the treatment [14]. Remarkably, the association of sulfasalazine and esomeprazole used in the present study was more efficacious, with 22% of the mice still alive off therapy after four months from the detection of the sarcoma, in the face of 100% of the untreated mice that died within 60 days. Although treated for two months with high doses of esomeprazole or esomeprazole plus sulfasalazine (corresponding to 2–5 times the maximal safe dose of esomeprazole given intravenously in human studies [51] and of sulfasalazine normally used in clinics [52]), mice that survived the MCA-induced sarcoma were healthy and displayed a normal behavior.

The double treatment also strongly decreased the number of TAM displaying the M2 marker CD206, which heavily infiltrate 3-MCA sarcoma from untreated mice, whereas the few CD86^+^ TAM present were unaffected by the therapy. Differently, in a murine T cell lymphoma growing in ascitic form, PPI administration resulted in enhanced recruitment TAM displaying M1 markers [53]. This apparent discrepancy may by due to differences in the microenvironment of solid and liquid tumors, which may generate TAM with different phenotypes [28, 54, 55], and to different sensitivity to M1 and M2 macrophages to esomeprazole and sulfasalazine as we have observed in the 3-MCA sarcoma.

The decreased number of M2 TAM in esomeprazole plus sulfasalazine -treated sarcoma may depend on a direct effect of the drugs on TAM or be secondary to their effects on tumor cells. The former possibility is supported by the increasing evidence that the molecules targeted by the two drugs are present also on myeloid cells: monocytes, macrophages and DCs upregulate xCT as an antioxidant system [29] and express membrane v-ATPases [30, 31] most likely as a consequence of the metabolic reprogramming to glycolysis [32]. Although the shift to glycolysis is described in M1 rather than M2 macrophages, our data show that v-ATPases are highly expressed on TAM displaying either M1 or M2 markers. This feature may depend on abnormal stimuli from the tumor microenvironment that drives a macrophages polarization different from that occurring in non-neoplastic inflamed or regenerating tissues. However, we observed that *in vitro* polarization of BMDM to the M2 phenotype is dramatically impaired by the combined exposure to sulfasalazine and esomeprazole, further supporting a direct effect of the two drugs on macrophages and suggesting that at least in some phases of polarization toward the M2 phenotype also normal macrophages express molecules targeted by the two drugs.

Low pH has been shown to inhibit tumor infiltrating T cells [56], and treatment with PPi to restore T cells function [57]. Thus, an additional mechanism for the antitumor activity of esomeprazole plus sulfasalazine might be through the restoration of local adaptive immunity against cancer. The observation that TAM displaying M1 phenotype are resistant to the double treatment supports this possibility. Whatever the mechanism, the concomitant decrease of tumor cell proliferation and migration and of M2 infiltration induced by esomeprazole plus sulfasalazine is likely to significantly increase the therapeutic efficacy of the combined therapy.

The improved antitumor efficacy obtained by associating esomeprazole and sulfasalazine is in part an additive effect, as they target two features of the tumor microenvironment -acidosis and reducing potential- both promoting tumor progression. In addition, the tight entanglement and the reciprocal potentiation of acidosis and reducing potential may explain the synergic effect of esomeprazole and sulfasalazine observed in some experiments. In fact, acidosis increases the reducing state of the microenvironment by triggering the production of ROS that causes overexpression of antioxidant genes, including xc- [49]. In turn, xc- overexpression may lower the extracellular pH by increasing the release of glutamic acid in exchange with cystine. Therefore, by blocking H^+^ extrusion, esomeprazole also contributes to the amelioration of the redox distress, whereas sulfasalazine, blocking xc-, not only inhibits the release of an antioxidant amino acid (cysteine), but also of an acidic one (glutamic acid). The synergy shown by the two drugs may be due also to the redox-dependency of esomeprazole-mediated v-ATPase inhibition: esomeprazole blocks v-ATPases by covalent bonding at a cysteine residue, a reaction counteracted by GSH that restores the activity of the inhibited proton pump[26]. The concomitant presence of sulfasalazine that, blocking xCT, decreases GSH levels [13] is likely to enhance the inhibition of v-ATPase by esomeprazole. Supporting the cooperation between the two drugs, the double treatment is more efficacious than esomeprazole in modulating pH. Also the induction of v-ATPase membrane expression, likely secondary to the drug-induced pHi decrease, is stronger in neoplastic cells treated with esomeprazole plus sCPG than in cells exposed to esomeprazole alone.

In conclusion, we have targeted two mechanisms of defense of tumor cells that not only preserve them from dying but also help tumor progression by using two drugs already in clinics, devoid of adverse effects, affordable and available in all countries, and found that their association can cure tumors.

It is well known that the gap between biomedical researchers and the patients who need their discoveries is wide, and difficult to close [58] due in part to the high cost and protracted time line of new drug discovery and development. The screening of existing drugs for new activities, in this case against tumors, is then a valuable short-cut between the lab and the clinic. If exploited more deeply this approach may provide good drugs, safe and effective at low prize [59].

## MATERIALS AND METHODS

### Reagents and antibodies

The following reagents and Antibodies were used: Esomeprazole, 3-methylcholanthrene, DTNB, Cristal violet, Sulfasalazine and LPS (Sigma-Aldrich); sCPG (Tocris Bioscience); LysoSensor Green DND-189, BCECF-AM and CFSE (Thermo Fisher Scientific);Cisplatin (Accord Healthcare); Mouse IFN-γ, IL-4 and recombinant GM-CSF (Relia Tech GmbH), rabbit anti-human xCT, mAbs anti-human Cytokeratin 14 (LL002) and Cytokeratin 18 (LDK 18) (Abcam); rabbit anti v-ATPase (TCIRG1, Proteintech); mAb anti-human thioredoxin (clone 2B1) kindly provided by Dr F. Clarke (University of Brisbane, Australia); mAbs to human Vimentin (V9) and Smooth Muscle Actin (1A4) (Thermofisher); rat anti-mouse CD206, CD86 (AbD Serotec), F4/80, CD86-FITC, F4/80-FITC (Biolegend) and CD206-FITC (Bio-Rad Laboratories).

### Primary tumor samples, tumor cell lines and culture

Human sarcoma and melanoma tissue samples were obtained from 10 patients upon approval of the institutional bioethics board and informed written consent of the patients [14]. The murine sarcoma cell line MCA was generated in 2015 in our laboratory from 3-MCA induced mouse sarcomas [14] by mechanical dissociation of tissues treated with 500 units/mL of collagenase type IA and 300 units/mL of hyaluronidase (Sigma-Aldrich). Sarcoma cell cultures were phenotypically characterized by immunostaining and found positive for Vimentin and Smooth Muscle Actin while negative for keratines [not shown, 60]. The murine melanoma cell lines B16-F10 was purchased from Interlab Cell Line Collection Biological in 2016. The human melanoma cell line MePa, kindly provided by Dr G. Pietra, was generated as reported [40]. A frozen stock was established immediately for each cell line, and only aliquots obtained from early cell passages were used in the study. Cell lines were routinely tested for mycoplasma contamination using a specific kit (Lonza).

### Determination of cell survival and proliferation

Cell viability and proliferation were determined by the crystal violet [13, 47] and the CFSE assays [42], respectively.

Dose-response experiments to determine the IC50 dose of esomeprazole and cisplatin at 96 hours of treatment have identified for esomeprazole 100μM (for all the cell lines used); for cisplatin: 0,3μM (for MCA), 2μM (for B16), 3μM (for MePa). sCPG and sulfasalazine were used at 300μM as previously determined [13, 41].

### Real-time PCR

Total RNA was isolated from cells by RNeasy mini kit (Qiagen, Milan, Italy) and reverse transcribed with the QuantiTect Reverse Transcription Kit (Qiagen), according to the manufacturer’s instructions. Real-time PCR was performed using SensiFAST™ SYBR (Bioline, Aurogene, Rome, Italy)

Primers were designed by PRIMER 3 (v.0.4.0) and purchased from TIB MOLBIOL (Genoa, Italy). RT-PCR conditions are described in Supplementary Table 1. Relative expression of target gene levels, normalized on the mean of hypoxanthine phosphoribosyltransferase 1 (HPRT) and β-actin housekeeping genes, was calculated by Q-Gene program [61].

### Staining procedures and immunohistochemistry

Serial cryostat sections from human and mice tumor samples, immediately snap frozen in OCT after removal, were stained with hematoxylin and eosin and processed for immunohistochemistry as described [3]. Cell counting was carried out in 8–12 randomly chosen fields independently by three researchers in a blind fashion; images were acquired with Leica DM RX microscopy using Scion Image software. Sections were stained with 1μM LysoSensor Green immediately after cutting. The specificity control was obtained by pre-incubating serial cryostat sections with buffer at pH 8.8 for 10 min before Lysosensor staining. Images were analyzed by confocal microscopy, acquired with Fluoview FV500 software (Olympus BioSystems) and the fluorescence was quantified using ImageJ software.

### Quantification of cysteine and lactate in culture media

For cysteine quantification, cell supernatants were reacted with 10mM DTNB and the absorption measured at 412 nm, as described [3]. For lactate, a colorimetric L-Lactate assay kit (Sigma) was used.

### pHi and pHe determination

Cell lines were loaded with 1μM BCECF-AM 30 min at 37°C. pHi was estimated as the ratio of the fluorescence signal obtained at 490 nm (pH sensitive) and 440 nm (isosbestic point) with the emission at 535 nm. Measurements were recorded by spectrofluorimetry (Spectra Max Gemini XPS, Molecular Devices). For pHe measurements, cells were seeded in triplicate and grown for 15h in medium containing 50% RPMI without NaHCO_3_, 50% medium with NaHCO_3_, 1% Nutridoma-SP (Roche), then treated 48 hours with or without drugs. pHe was measured at 25°C using a Jenco 6230N pHmeter.

### Flow cytometric analyses

Surface expression of vATPase was detected by flow cytometry (CyAn, Beckman Coulter) using anti-vATPase Ab and analyzed by Summit V4.3 software. Background fluorescence was set on untreated and unstained cells.

### Gap filling assay

Cell lines, plated at 10^5^/wells in 6 well plates and cultured 48 hours with or without esomeprazole, sCPG or both, were harvested, counted and plated in a μ-dish, 35mm, low, culture-insert plate in medium alone or with drugs (Ibidi). In these plates, a silicone dam of 500 μm separates the plating space in two identical wells in which identical number of cells (60×10^3^) were let adhere for 6 hours. After that period, dams were removed leaving a defined cell-free gap and time-lapse 10X photographs of gap closure were taken each 5 min for up to 24 hours using a Zeiss Axiovert S100 TV2 microscope (Zeiss) equipped with a Hamamatsu OrcaII-ER camera (Hamamatsu City,) and analyzed using ImageJ or Oko-vision (Okolab) softwares [47]. Similar experiments were repeated using sulfasalazine in place of sCPG.

### Mice

Balb/C and C57Bl/6J mice of 8-10 week old (Envigo) were bred and kept under germ-poor conditions at the animal facility of the IRCCS AOU San Martino-IST. All mouse studies were approved by the Institutional Animal Care and were cared for in accordance with national legislative provisions for the protection of animals used for scientific purposes.

### Syngenic tumor transplant and 3-MCA-induced mouse carcinogenesis

In the syngenic transplantation model, MCA (0,2×10^6^) and B16 (0,2×10^6^) cells were subcutaneously (s.c.) implanted in the hind flank of immunocompetent syngeneic Balb/C and C57Bl/6J mice, respectively. Tumor development was monitored every day.

In the 3-MCA-induced carcinogenesis, Balb/C mice were s.c. injected in the hind flank with 500 μg of 3-MCA suspended in 0,1 ml of olive oil [14]. Tumor development was monitored 2-3 times weekly for 4-5 months.

The tumor volume was determined using the following formula: d^2^ x D x 0,52, where d and D are the short and long dimension (cm) of the tumor, respectively, measured with a caliper [14]. Mice with a tumor ≥ 0,3 cm in diameter were counted as tumor positive and began the treatments. Euthanasia was performed when the tumors reached a volume of about 1,5 cm^3^.

### Protocols of *in vivo* treatments

In syngenic tumor transplants, 12 mice per group were untreated or treated with esomeprazole, sulfasalazine or esomeprazole plus sulfasalazine starting the treatment 5 hours after cell injection. Four mice per group were untreated or treated with the same drugs starting when the tumors reached ≥ 0,3 cm in diameter (5 days after cell injection).

In 3-MCA-induced tumorigenesis 15 mice per group were untreated or treated for 60 days as above starting when the tumor became palpable.

Esomeprazole was provided 3 times for week intraperitoneally (i.p.) at 12,5mg/kg in 200μl saline [62]; sulfasalazine was provided daily i.p. at 250mg/kg in 400μl as described [14]; when the two drugs were given in association they were spaced by at least 6 hours.

Untreated mice received saline only.

### Generation of M2 macrophages *in vitro*

To generate BMDM, the bone marrow cells from femurs and tibias from mice were harvested and cultured as previously described [63]. Briefly, isolated cells were incubated in RPMI1640 supplemented with 10% FBS plus mouse GM-CSF, 50ng/ml. On day 7, cells were washed, counted and re-plated in the same media without GM-CSF at a density of 0,5 X10^6^ cells. On day 8, cells were alternatively activated (M2 condition) with IL-4 (20ng/ml) or received media alone (M0 condition). Polarization was carried out in the absence or presence of esomeprazole, sCPG or esomeprazole plus sCPG. Cells were harvested after 48 hours and stained with Ab directed to markers of mature macrophage (anti-F4/80), M1 (anti-CD86) and M2 (anti-CD206) macrophages by flow cytometry or fixed for immunohystochemical assays.

### Statistical analysis

Statistical analyses were done by T-test or one-way ANOVA test, followed by Bonferroni post-test, as appropriate. Survival rate was performed by Kaplan–Meier analysis and compared by the Mantel–Cox test. All statistical tests were carried out using GraphPad Prism (version 4.0). Significance was assumed at P < 0.05.

## SUPPLEMENTARY MATERIALS FIGURES AND TABLE



## Abbreviations

3-MCA: 3-methylcholanthrene
ESO: esomeprazole
PPIs: proton pump inhibitors
SASP: sulfasalazine
sCPG: (S)-4 carboxyphenylglycine
TAM: tumor-associated macrophages
