# Supplementary material for: Restoring microenvironmental redox and pH homeostasis inhibits neoplastic cell growth and migration: therapeutic efficacy of esomeprazole plus sulfasalazine on 3-MCA-induced sarcoma
Source: Oncotarget. 2017 Jun 27;8(40):67482–96. doi: 10.18632/oncotarget.18713 (PMC5620187; doi:10.18632/oncotarget.18713)
Supplement: Supplementary file 1 [file oncotarget-08-67482-s001.pdf]

# Restoring microenvironmental redox and pH homeostasis inhibits neoplastic cell growth and migration: therapeutic efficacy of esomeprazole plus sulfasalazine on 3-MCA-induced sarcoma

## SUPPLEMENTARY MATERIALS

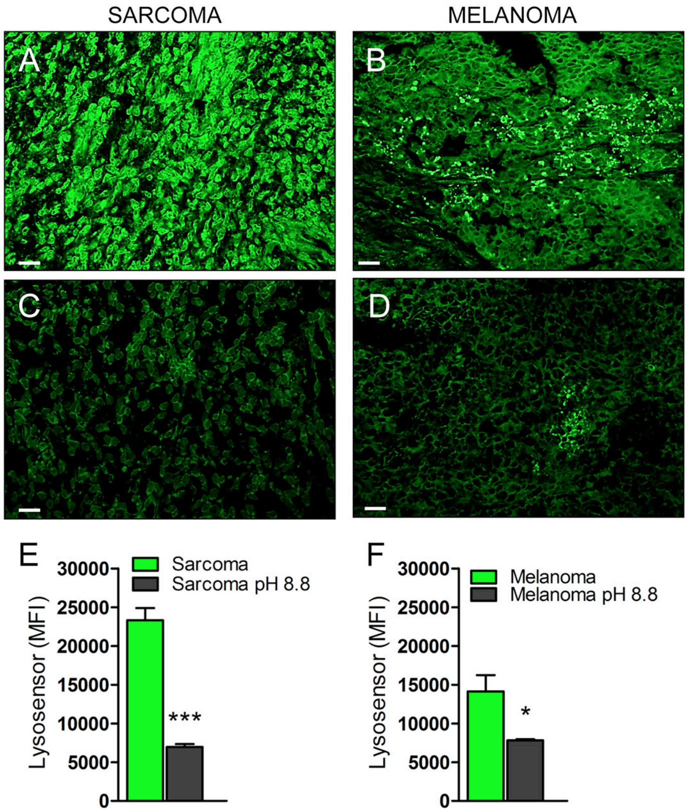


**Supplementary Figure 1: Specificity of the Lysosensor staining.** The serial cryostat sections of a human sarcoma **(A,C)** and a human melanoma **(B,D)** were stained with Lysosensor green. In **(C)** and **(D)** the section were pre-incubated in buffer at pH 8.8 for 10 min before staining. **(E-F)** Quantification of the LysoSensor fluorescence levels in sarcoma **(A-C)** and melanoma **(B-D)** is indicated. Results are expressed as mean fluorescence intensity (MFI) obtained in 20 chosen fields ± SEM. *P < 0.05; ***P < 0.001. Scale bar, 30µm.

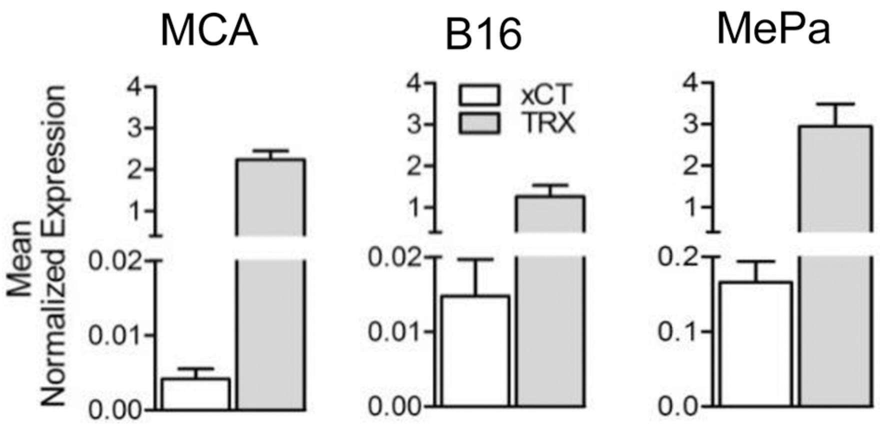


**Supplementary Figure 2: mRNA expression of xCT and Thioredoxin.** Real Time PCR analysis of xCT (Slc7a11) and Thioredoxin (TRX) mRNA expression in MCA, B16 and MePa cells. The results are plotted as the Mean of the Normalized Expression vs housekeeping genes. (n = 2 ± SEM).

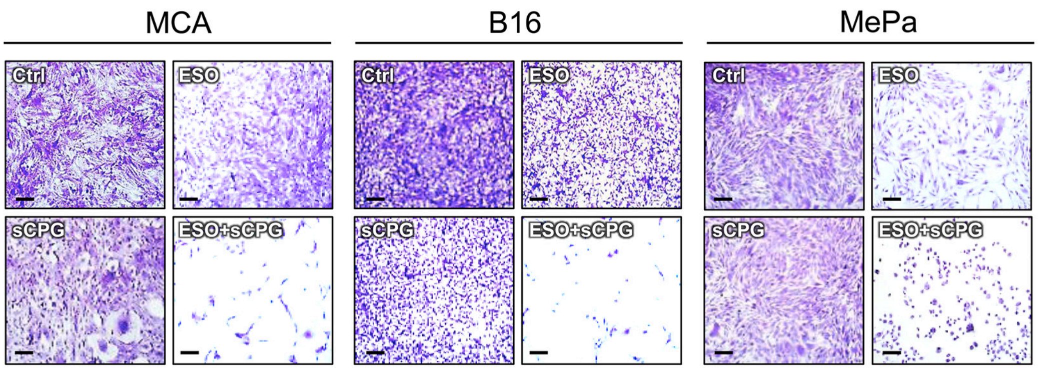


**Supplementary Figure 3: Esomeprazole and sCPG impair the tumor cell survival.** Representative images of crystal violet staining at 96 h of MCA, B16 and MePa cells untreated or treated with ESO and sCPG, alone or in combination. Scale bar, 30µm.

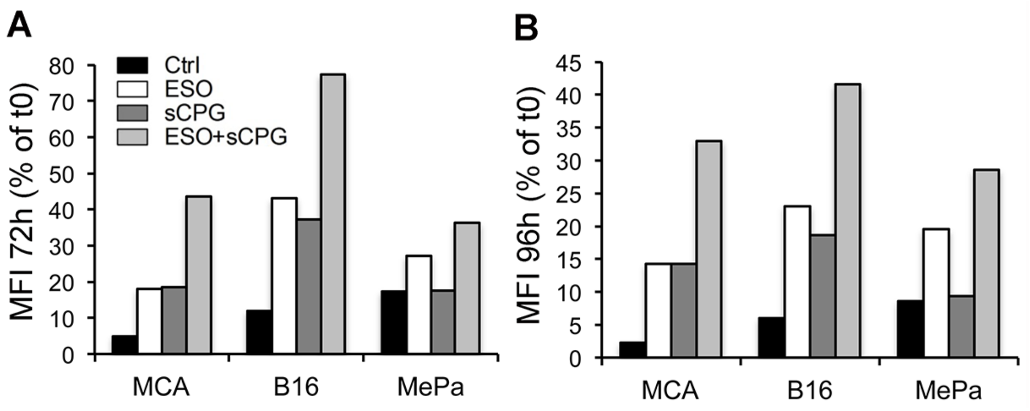


**Supplementary Figure 4: Esomeprazole and sCPG impair tumor cell proliferation.** MCA, B16 and MePa cells were loaded with CFSE and untreated (Ctrl) or treated with ESO and sCPG, alone or in combination. CFSE fluorescence is expressed as percentage of MFI of t 0 at 72 h and 96 h.

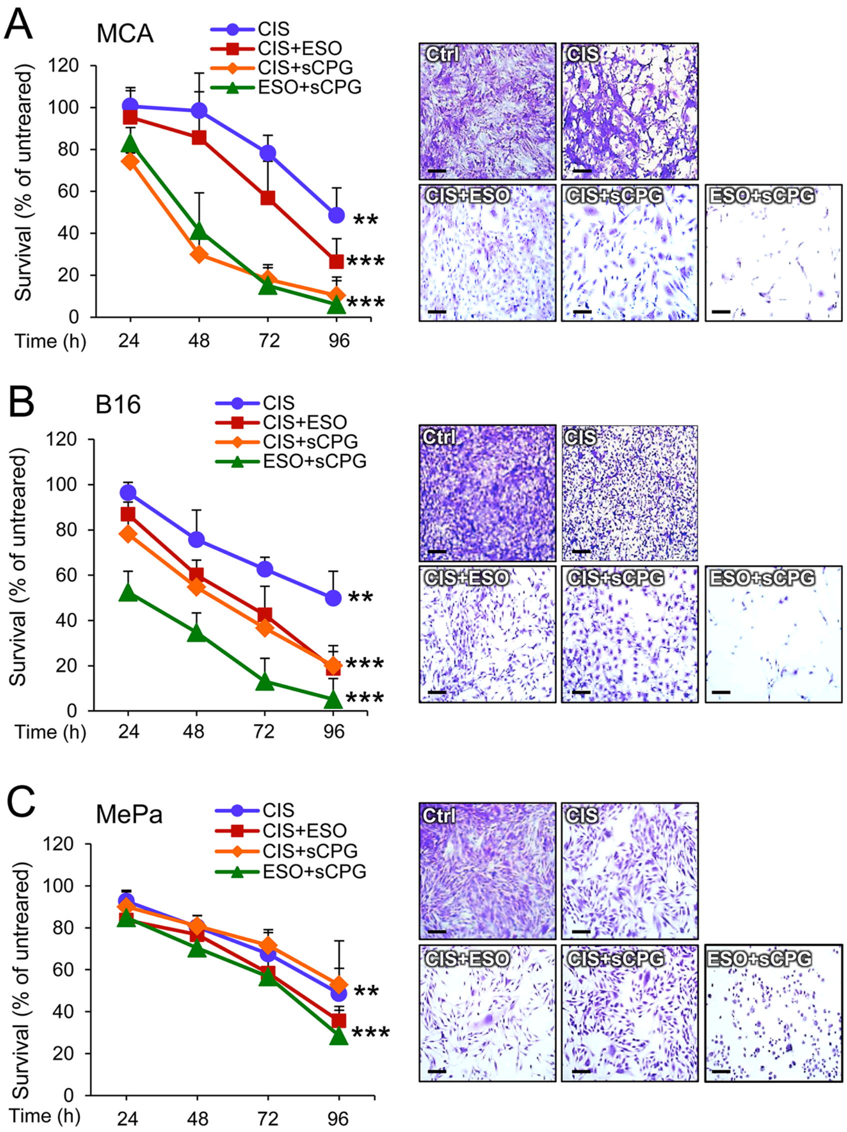


**Supplementary Figure 5: Esomeprazole plus sCPG is more efficacious in impairing tumor cell survival than Cisplatin alone.** Survival rate of MCA **(A)** B16 **(B)** and MePa **(C)** cells treated with ESO plus sCPG and with Cisplatin alone or in combination with ESO or sCPG, for 24 h, 48 h, 72 h and 96 h was determined by Cristal Violet assay. Data are expressed as percent of control untreated cells. Representative images of crystal violet staining at 96 h are shown in adjacent panels. Scale bar, 30μm. Statistical analysis was performed vs control untreated cells **P < 0.01, ***P < 0.001.

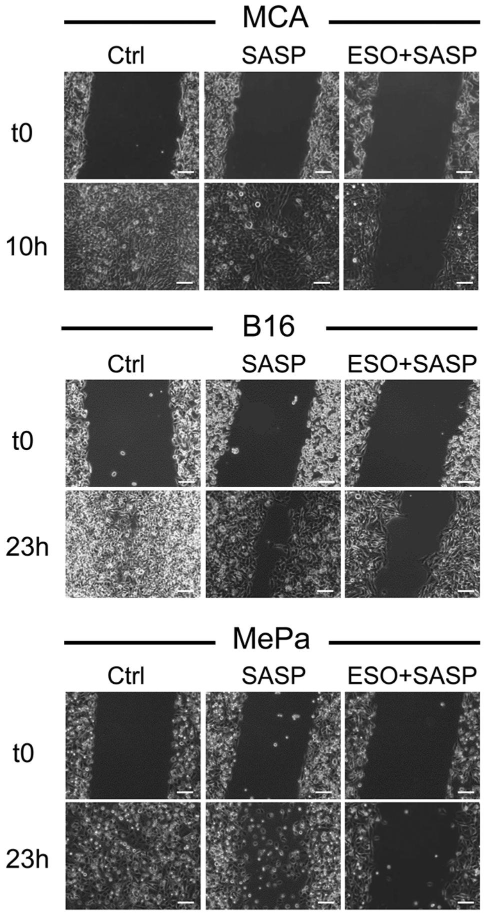


**Supplementary Figure 6: Esomeprazole plus SASP impair tumor cell growth, proliferation and migration.** Cells untreated (Ctrl) or pretreated 48 h with SASP alone or in combination with ESO were analyzed in a gap filling assay (see Materials and Methods). The images show frames of a representative video, taken after 10 h (MCA) or 23 h (B16 and MePa) of culture at 37°C. Scale bar corresponds to 200µm. One representative experiment out of 2 performed is shown.

**Supplementary Table 1: Primers and conditions for Real Time PCR**

| Gene (ID) | NCBI Reference Sequence | Primer sequences (5'-3') | Primer concentrations (µM/L) |
|---|---|---|---|
| m-xct (26570) | NM_011990.2 | F AAACCCAAGTGGTTCAGACG<br>R ATCTCAATCCTGGGCAGATG | 0.3<br>0.3 |
| m-trx1 (22166) | NM_011660.3 | F TCCAATGTGGTGTTCCTTGA<br>R CCTTGTTAGCACCGGAGAAC | 0.3<br>0.3 |
| m-hprt (15452) | NM_013556.2 | F CGTCGTGATTAGCGATGATG<br>R AGCAAGTCTTTCAGTCCTGTCC | 0.25<br>0.25 |
| m-actb (11461) | NM_007393.5 | F TTACTGCTCTGGCTCCTAGCAC<br>R GCTGGAAGGTGGACAGTGAG | 0.25<br>0.25 |
| h-xCT (23657) | NM_014331.3 | F GCTGGGCTGATTTATCTTCG<br>R AAAGCTGGGATGAACAGTGG | 0.3<br>0.3 |
| h-TRX1 (7295) | NM_003329.3 | F GCCTTTCTTTCATTCCCTCTC<br>R TCTGAAGCAACATCCTGACAGT | 0.3<br>0.3 |
| h-HPRT (3251) | NM_000194.2 | F CCTGGCGTCGTGATTAGTG<br>R ACACCCTTTCCAAATCCTCAG | 0.25<br>0.25 |
| h-ACTB (60) | NM_001101.3 | F TGCCCTGAGGCACTCTTC<br>R TGAAGGTAGTTTCGTGGATGC | 0.25<br>0.25 |

Gene ID from www.ncbi.nlm.nih.gov/gene; h : human; m: mouse; F: forward; R: reverse; Ta:; xCT (SLC7A11) : solute carrier family 7 member 11; TRX: Thioredoxin; HPRT: hypoxanthine phosphoribosyltransferase 1; ACTb : β actin; annealing temperature: 60°C

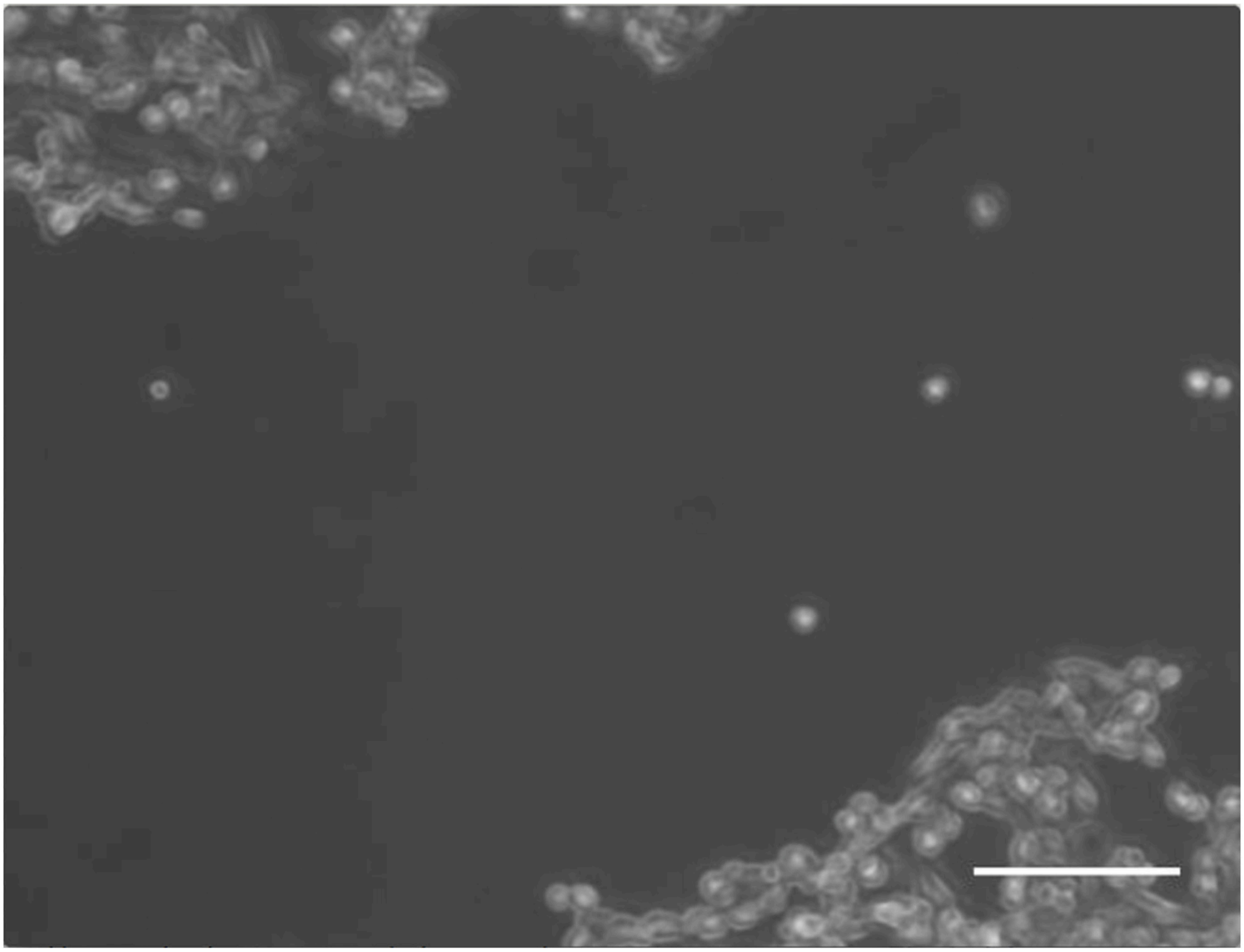

**Supplementary Videos 1: Gap-filling of MCA cells.** Representative video (n=4) showing the kinetics of gap closure by MCA cells untreated or treated with ESO and sCPG alone or in combination. Cells were filmed for up to 24 h although the gap was already closed at 16 h 40min. The scale bars correspond to 200µm.

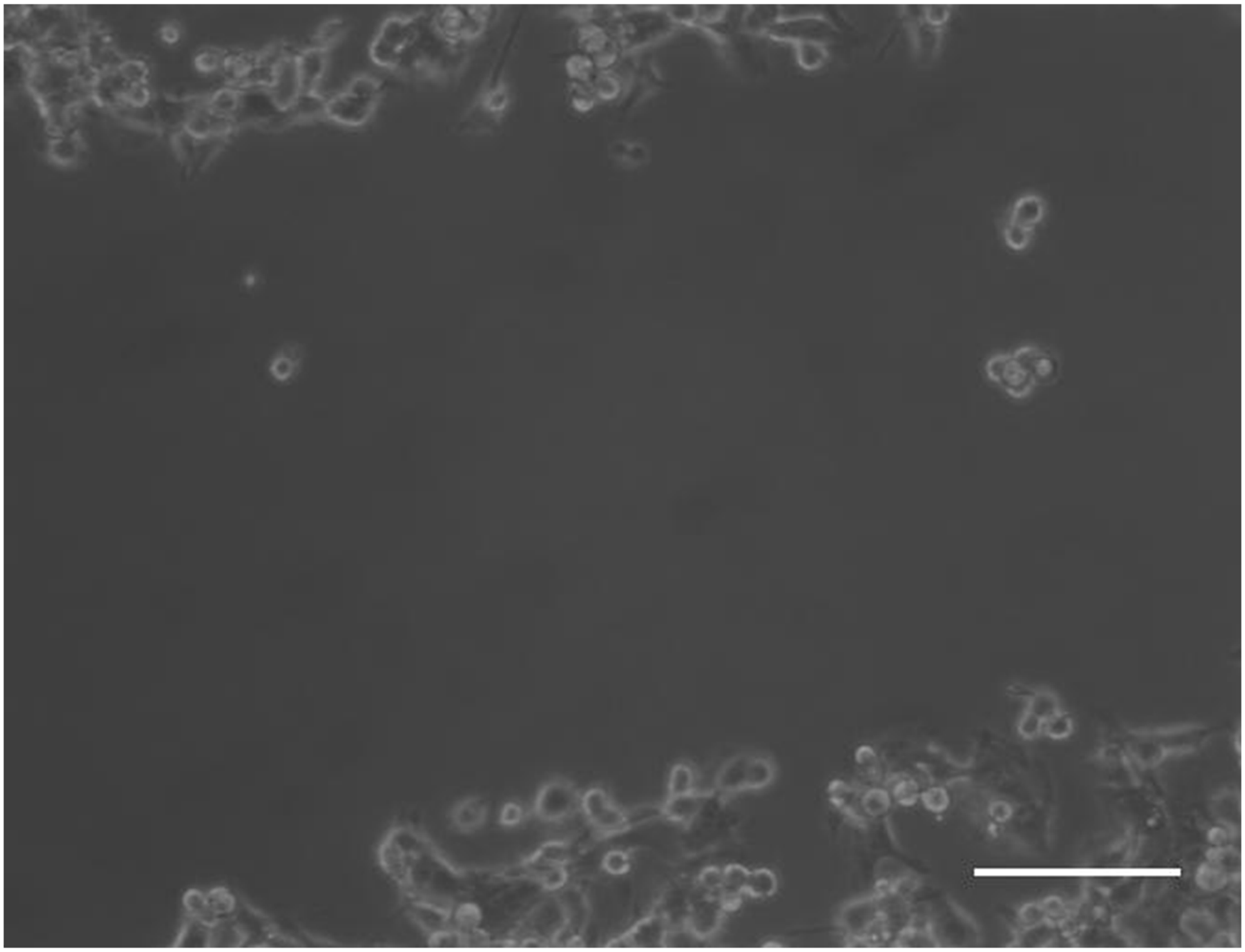

**Supplementary Videos 2: Gap-filling of MCA cells.** Representative video (n=4) showing the kinetics of gap closure by MCA cells untreated or treated with ESO and sCPG alone or in combination. Cells were filmed for up to 24 h although the gap was already closed at 16 h 40min. The scale bars correspond to 200µm.

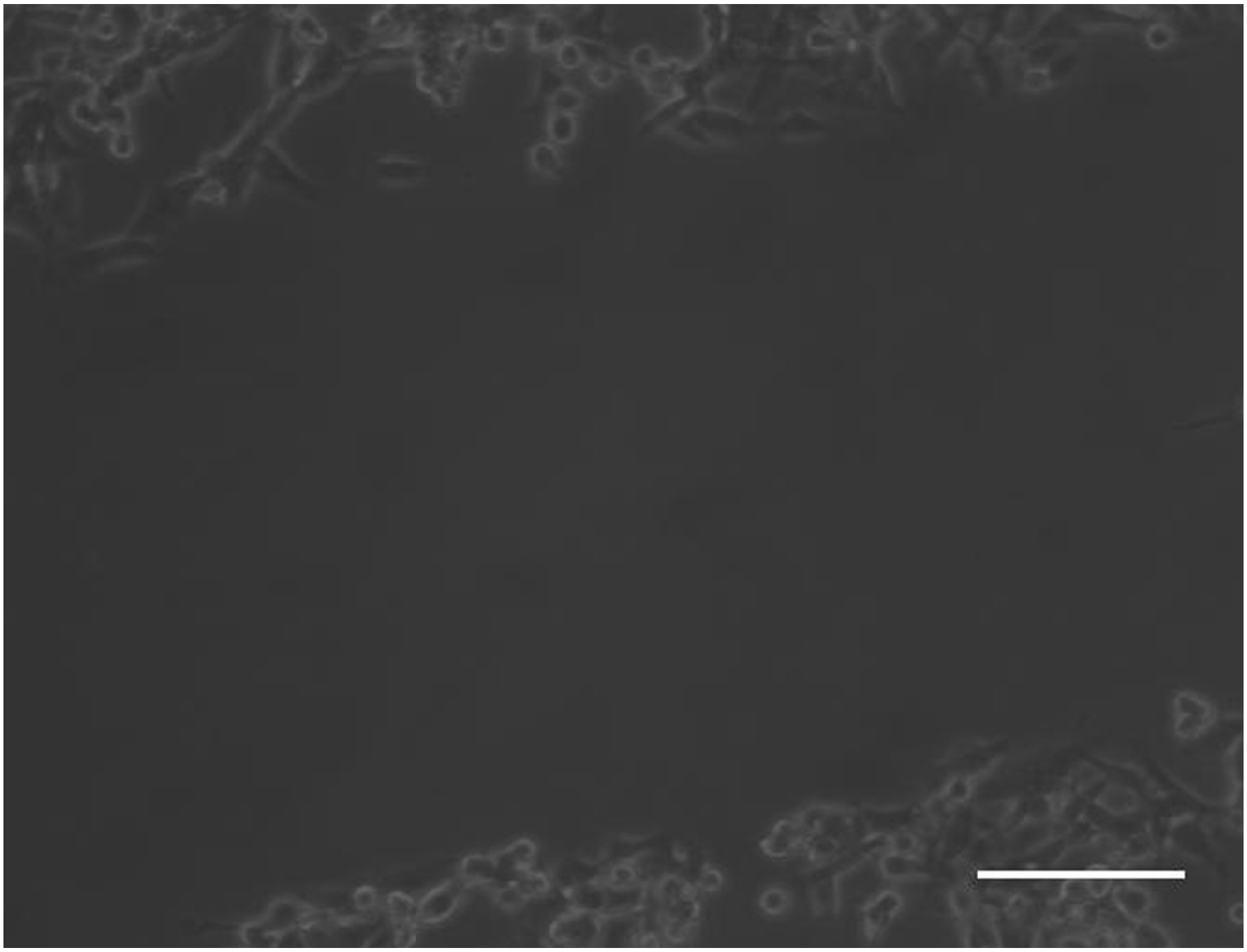

**Supplementary Videos 3: Gap-filling of MCA cells.** Representative video (n=4) showing the kinetics of gap closure by MCA cells untreated or treated with ESO and sCPG alone or in combination. Cells were filmed for up to 24 h although the gap was already closed at 16 h 40min. The scale bars correspond to 200µm.

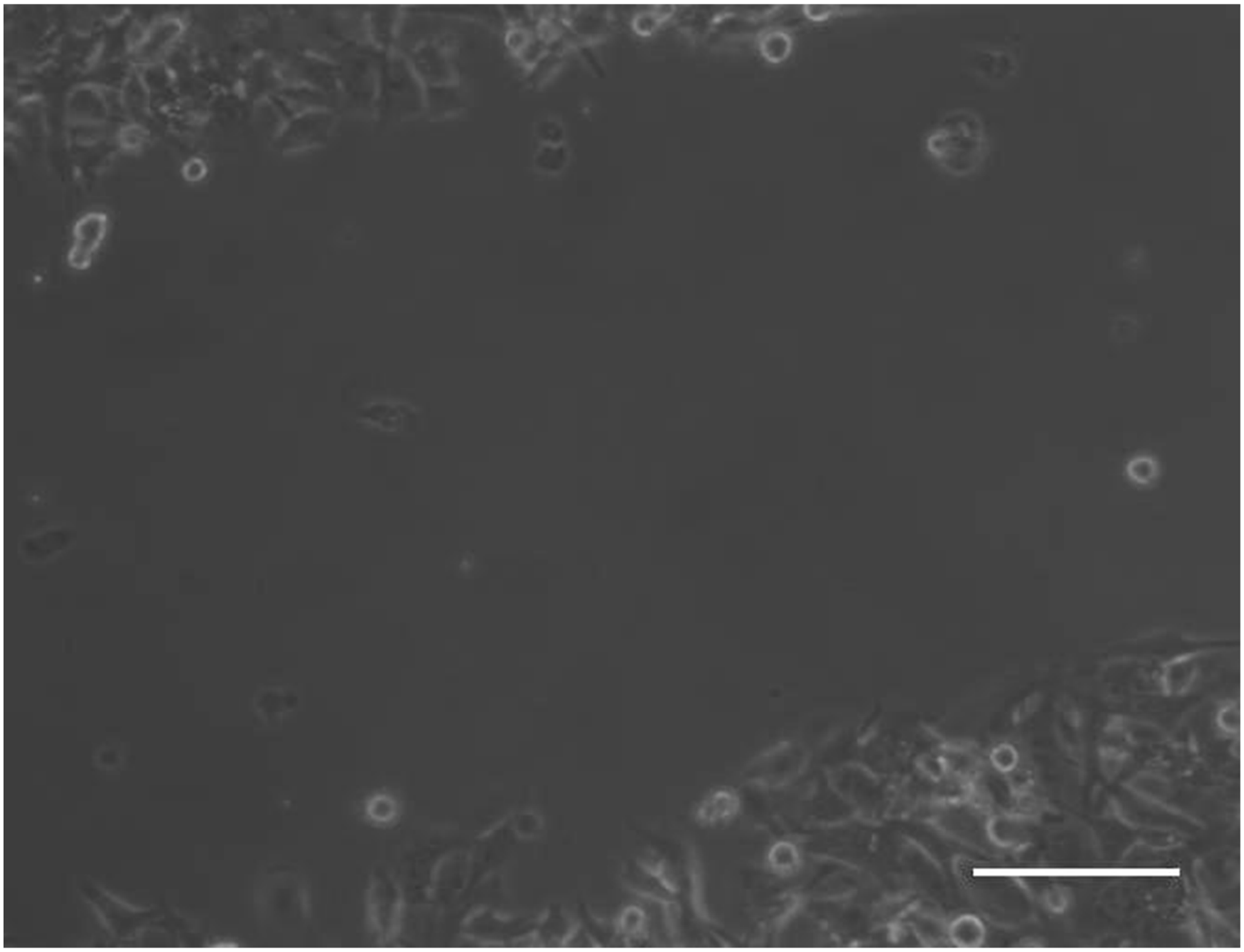

**Supplementary Videos 4: Gap-filling of MCA cells.** Representative video (n=4) showing the kinetics of gap closure by MCA cells untreated or treated with ESO and sCPG alone or in combination. Cells were filmed for up to 24 h although the gap was already closed at 16 h 40min. The scale bars correspond to 200µm.

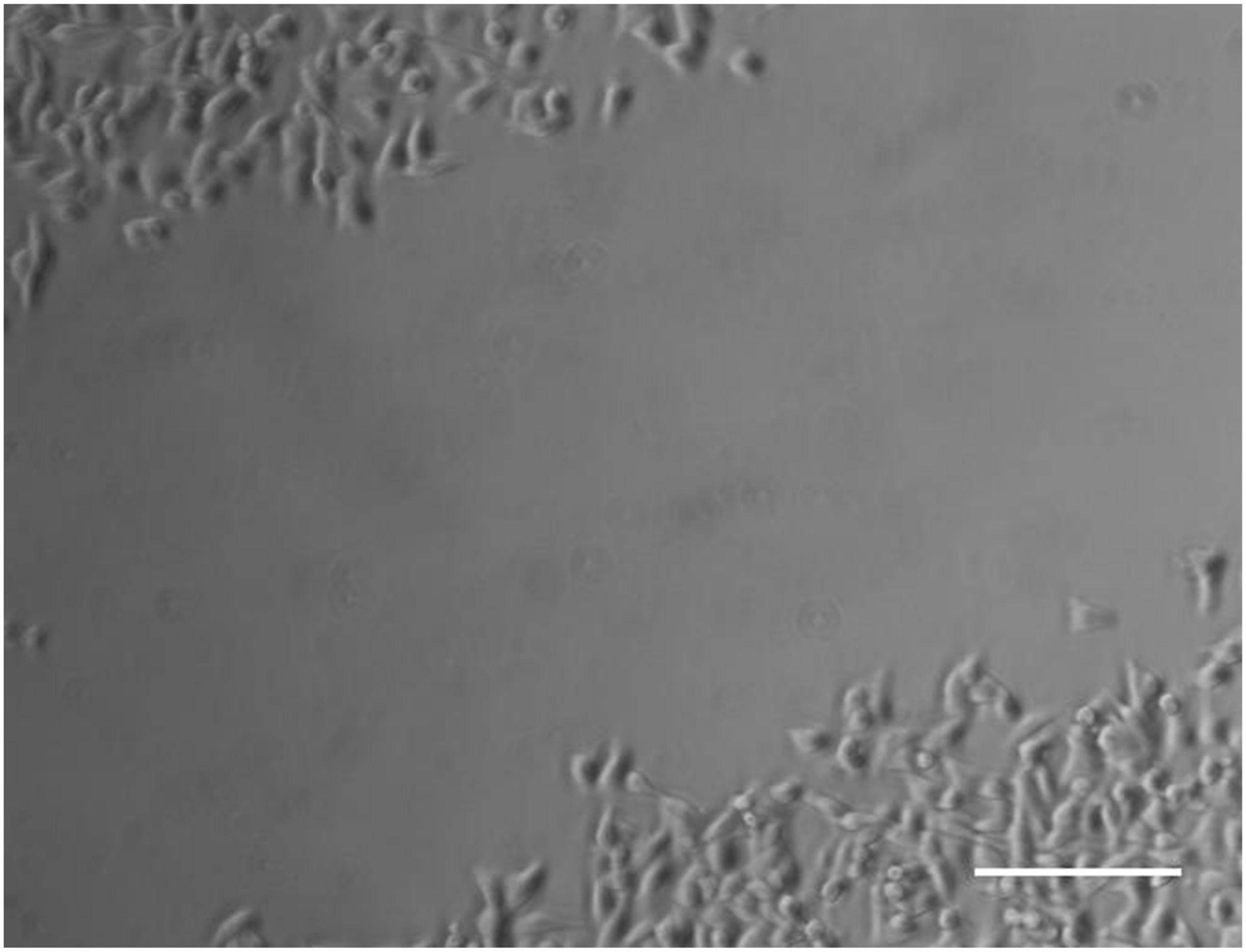

**Supplementary Videos 5: Gap-filling of B16 cells.** Representative video (n=4) showing the kinetics of gap closure by B16 cells untreated or treated with ESO and sCPG alone or in combination filmed for 24 h. The scale bars correspond to 200µm.

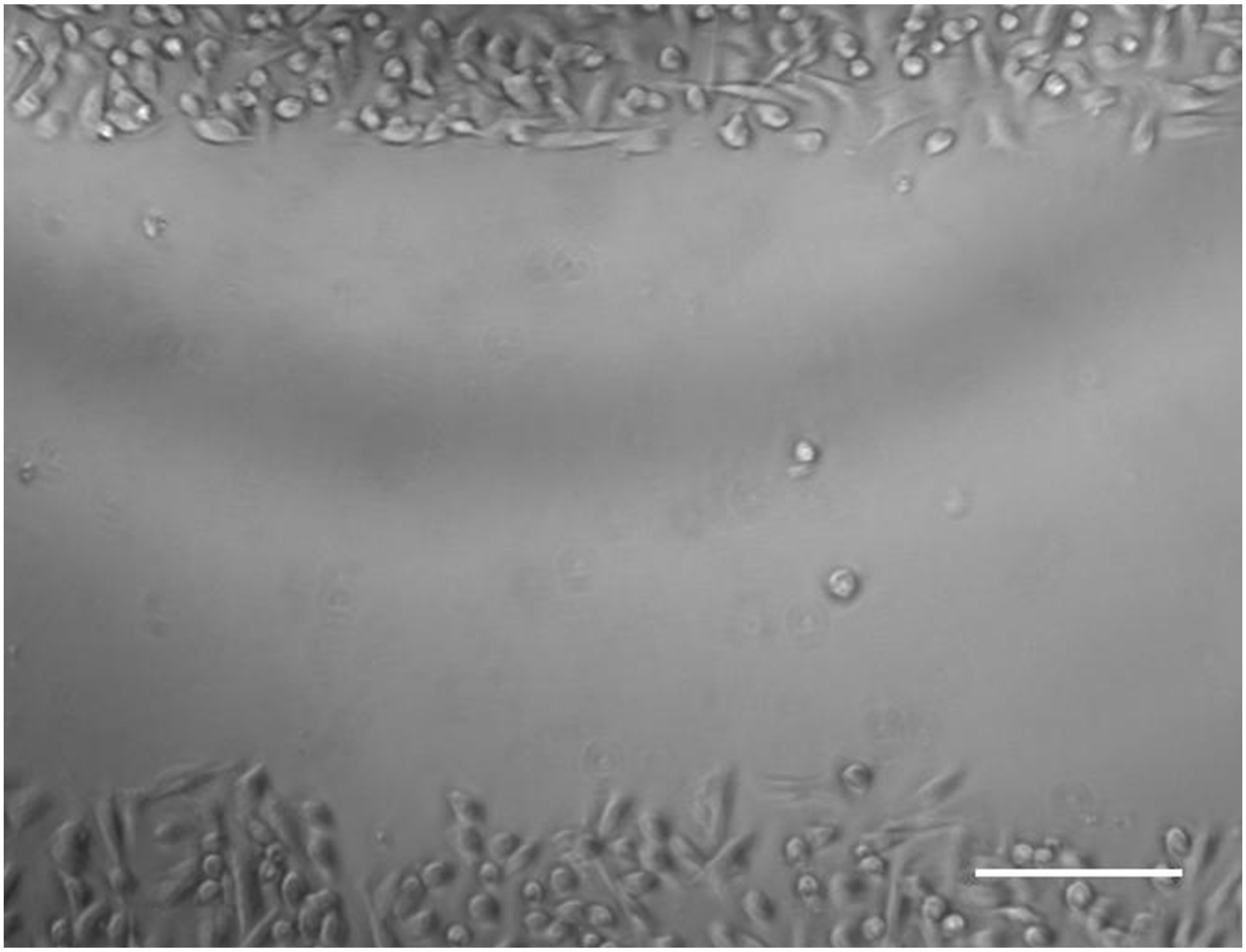

**Supplementary Videos 6: Gap-filling of B16 cells.** Representative video (n=4) showing the kinetics of gap closure by B16 cells untreated or treated with ESO and sCPG alone or in combination filmed for 24 h. The scale bars correspond to 200µm.

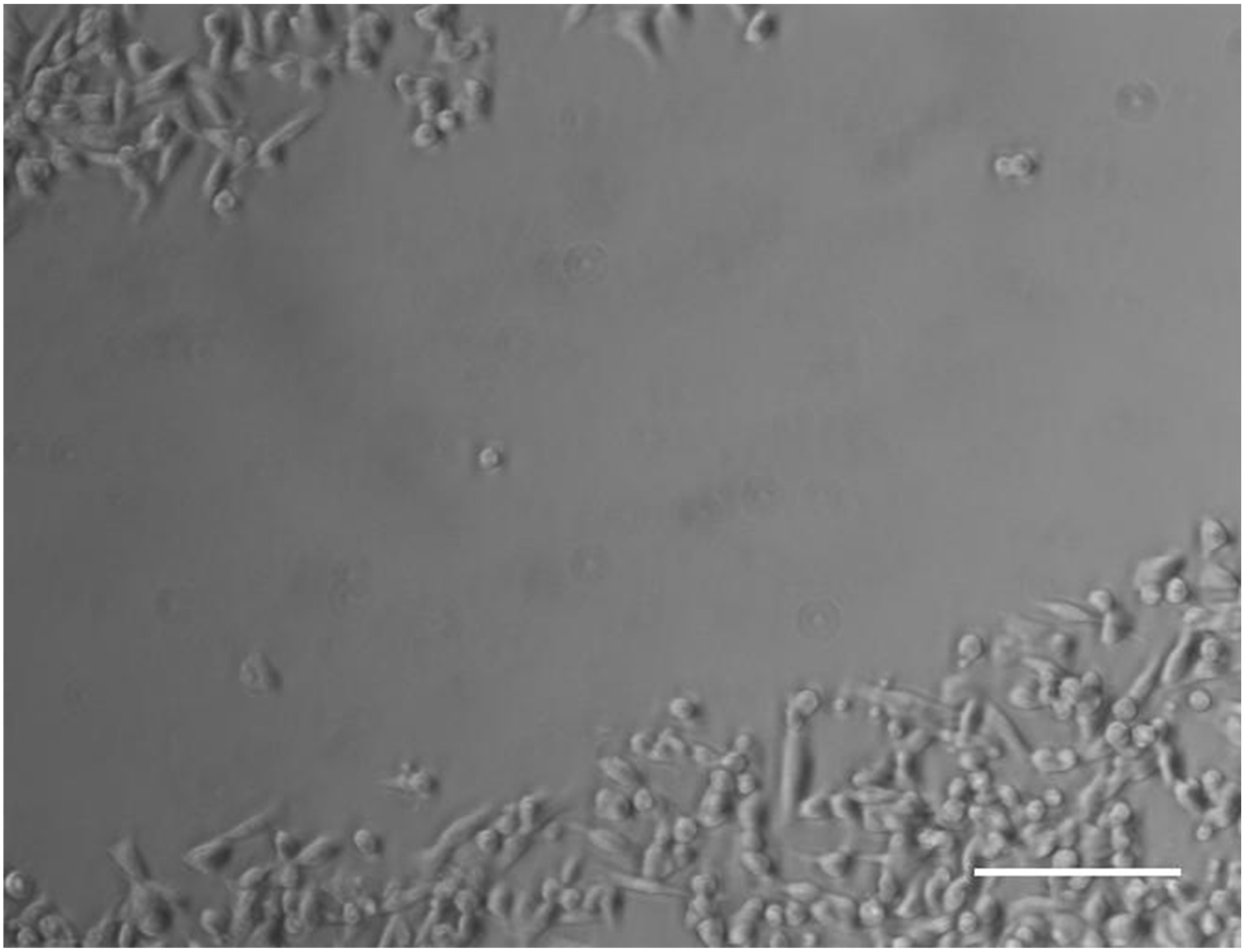

**Supplementary Videos 7: Gap-filling of B16 cells.** Representative video (n=4) showing the kinetics of gap closure by B16 cells untreated or treated with ESO and sCPG alone or in combination filmed for 24 h. The scale bars correspond to 200µm.

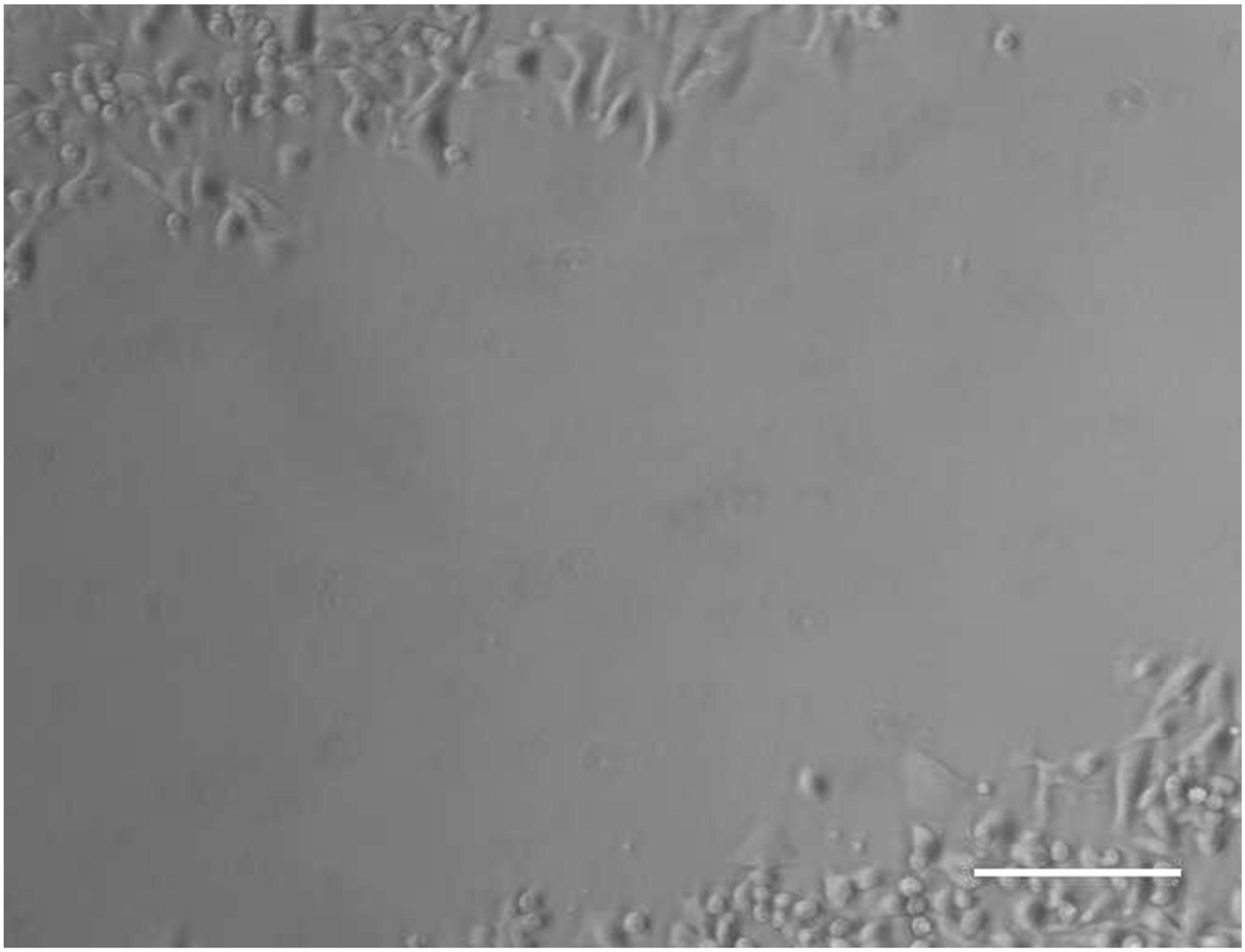

**Supplementary Videos 8: Gap-filling of B16 cells.** Representative video (n=4) showing the kinetics of gap closure by B16 cells untreated or treated with ESO and sCPG alone or in combination filmed for 24 h. The scale bars correspond to 200µm.

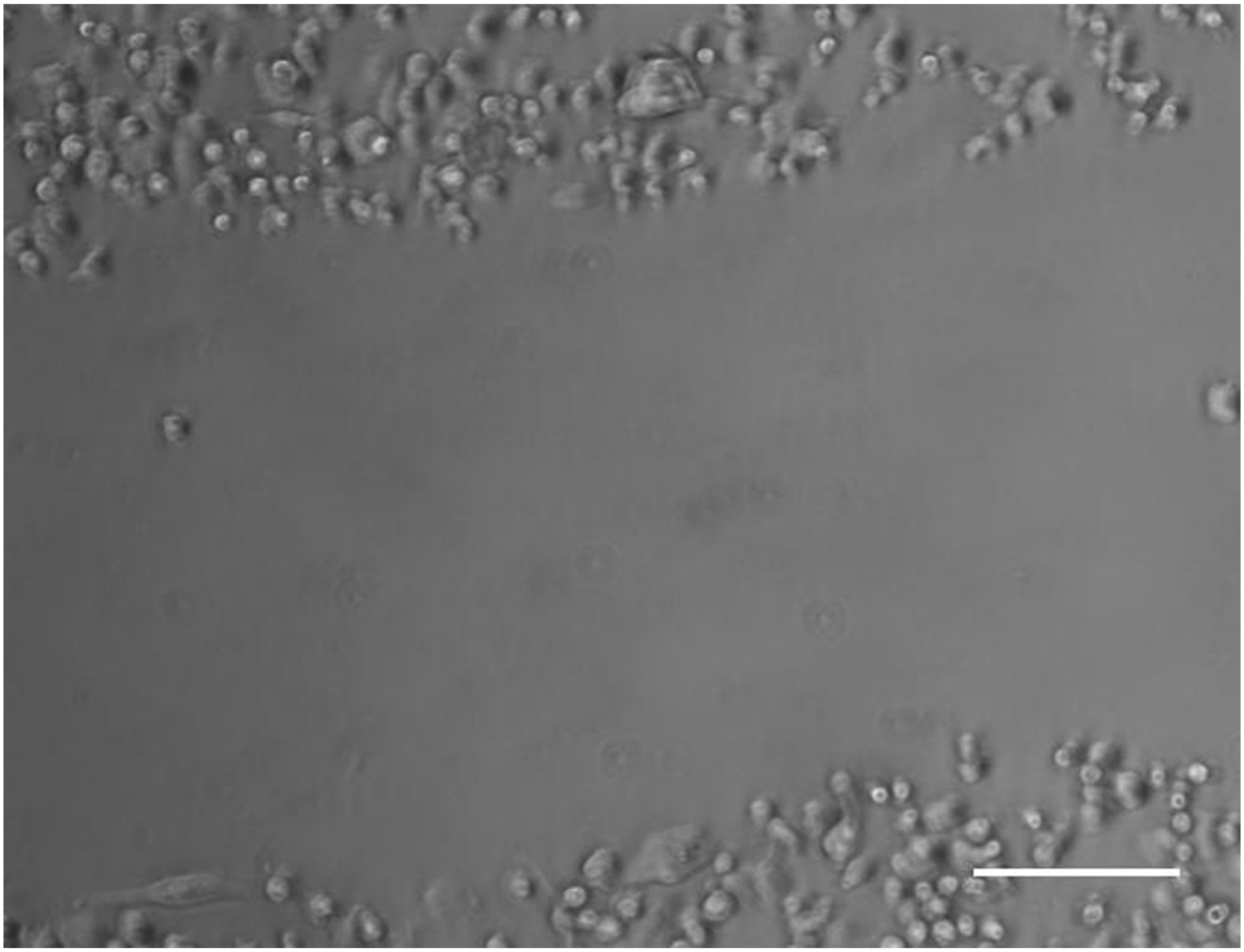

**Supplementary Videos 9: Gap-filling of MePa cells.** Representative video (n=4) showing the kinetics of gap closure by MePa cells untreated or treated with ESO and sCPG alone or in combination filmed for 24 h. The scale bars correspond to 200µm.

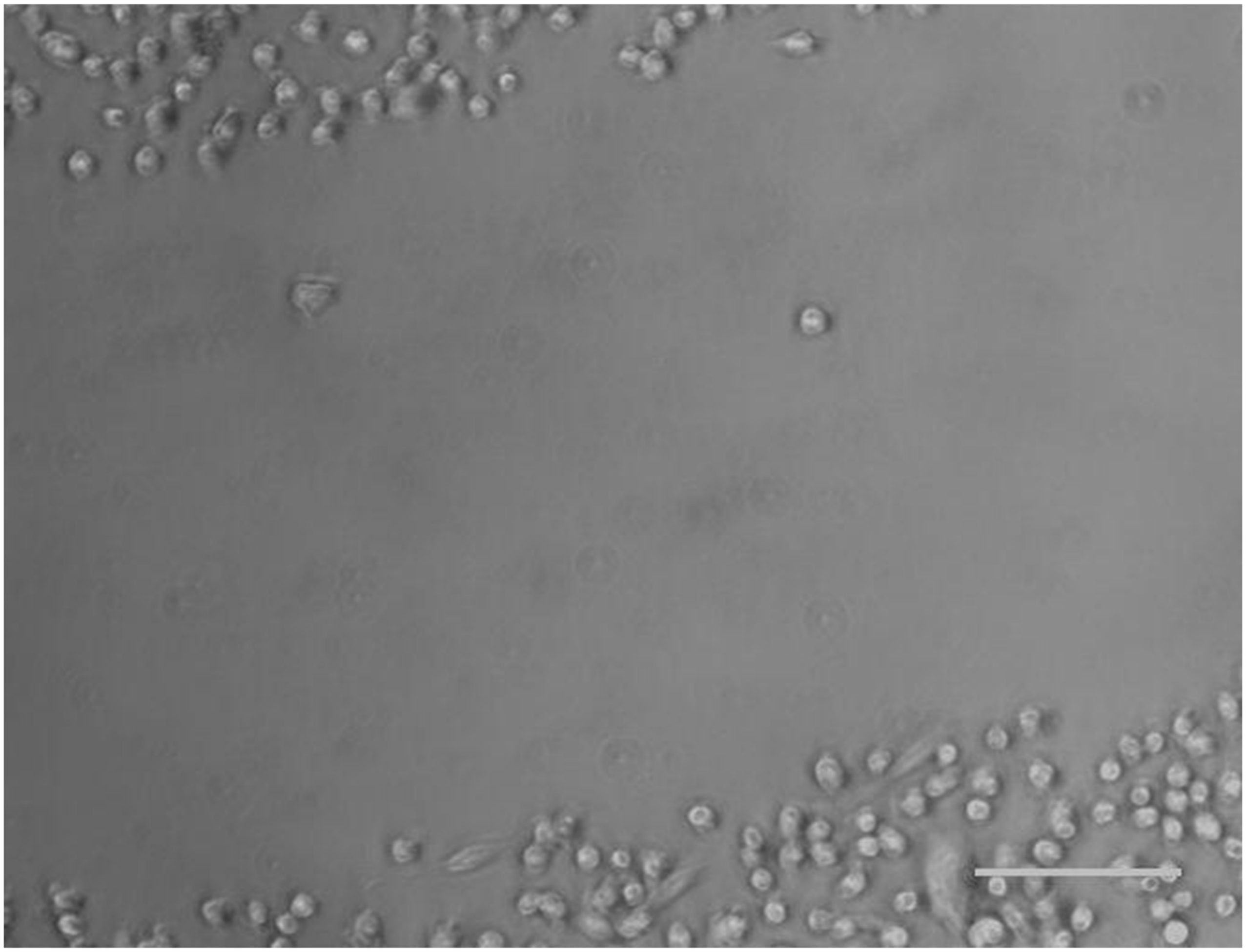

**Supplementary Videos 10: Gap-filling of MePa cells.** Representative video (n=4) showing the kinetics of gap closure by MePa cells untreated or treated with ESO and sCPG alone or in combination filmed for 24 h. The scale bars correspond to 200µm.

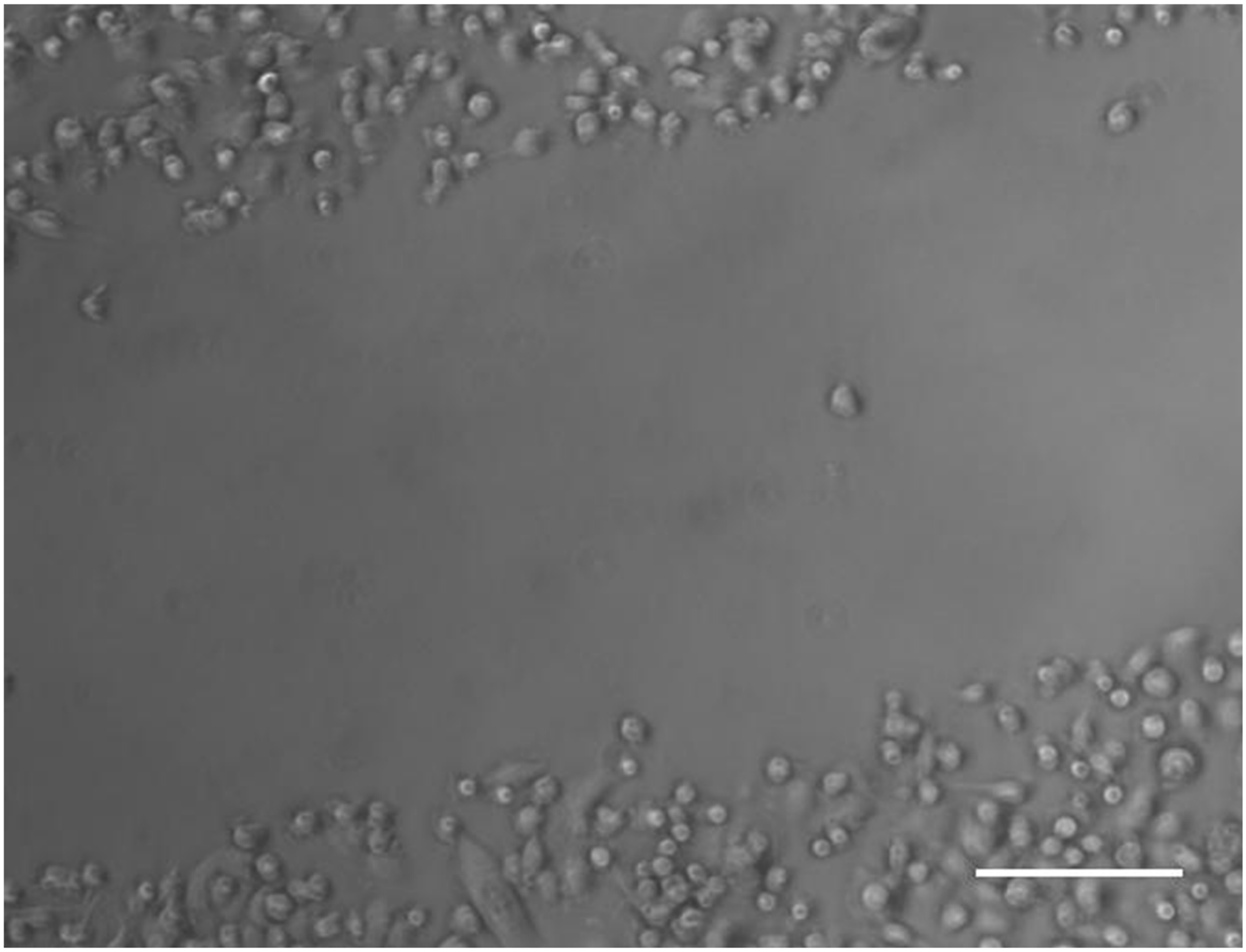

**Supplementary Videos 11: Gap-filling of MePa cells.** Representative video (n=4) showing the kinetics of gap closure by MePa cells untreated or treated with ESO and sCPG alone or in combination filmed for 24 h. The scale bars correspond to 200μm.

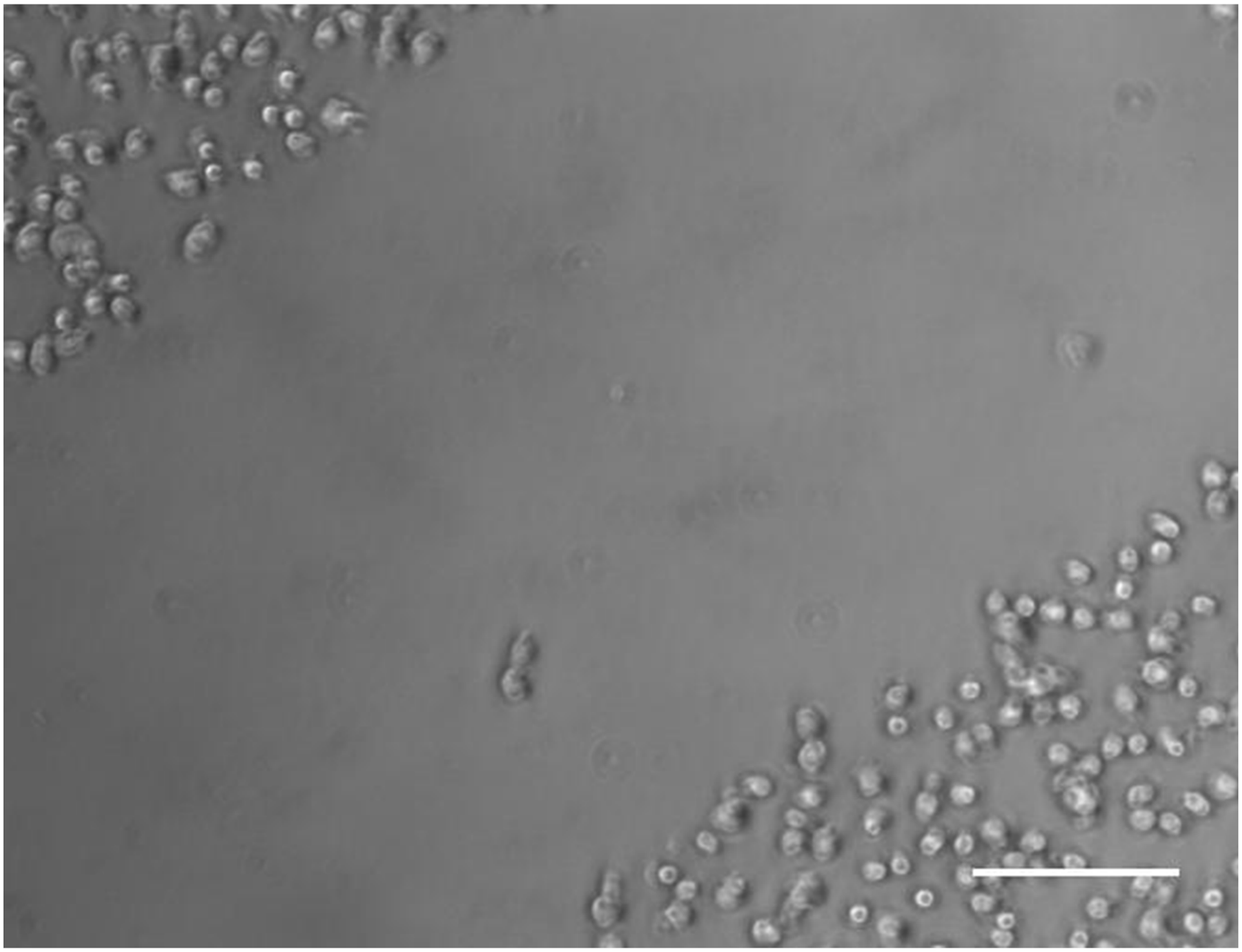

**Supplementary Videos 12: Gap-filling of MePa cells.** Representative video (n=4) showing the kinetics of gap closure by MePa cells untreated or treated with ESO and sCPG alone or in combination filmed for 24 h. The scale bars correspond to 200µm.
